# Army ant middens – Home and nursery of a diverse beetle fauna

**DOI:** 10.1002/ece3.10451

**Published:** 2023-09-19

**Authors:** Christoph von Beeren, Sebastian Pohl, Martin Fikáček, Stephan Kleinfelder, Alexey K. Tishechkin, Shûhei Yamamoto, Mariana Chani‐Posse, Dagmara Żyła, Alexandra Tokareva, Munetoshi Maruyama, W. Eugene Hall, Liliana P. Sandoval, Daniel J. C. Kronauer

**Affiliations:** ^1^ Department of Biology Technical University of Darmstadt Darmstadt Germany; ^2^ Laboratory of Social Evolution and Behavior The Rockefeller University New York New York USA; ^3^ NUS College National University of Singapore Singapore Singapore; ^4^ Division of Science Yale‐NUS College Singapore Singapore; ^5^ Department of Biological Sciences National Sun Yat‐sen University Kaohsiung Taiwan; ^6^ Department of Entomology National Museum Prague Czech Republic; ^7^ California State Collection of Arthropods California Department of Food and Agriculture Sacramento California USA; ^8^ The Hokkaido University Museum Hokkaido University Sapporo Japan; ^9^ Instituto Argentino de Investigaciones de las Zonas Aridas Mendoza Argentina; ^10^ Museum of Nature Hamburg Leibniz Institute for the Analysis of Biodiversity Change Hamburg Germany; ^11^ Museum and Institute of Zoology of the Polish Academy of Sciences Warsaw Poland; ^12^ The Kyushu University Museum Hakozaki Fukuoka Japan; ^13^ University of Arizona Insect Collection Tucson Arizona USA; ^14^ Department of Ecology, Faculty of Environmental Sciences Czech University of Life Sciences Prague Praha — Suchdol Czech Republic; ^15^ Howard Hughes Medical Institute New York New York USA

**Keywords:** army ant, biodiversity, Coleoptera, DNA barcoding, scavenger, tropical rainforest

## Abstract

Army ants provide nourishment to a large variety of animals. This includes birds that feed on animals flushed out by army ant raids, symbiotic arthropods that consume the ants' prey or their brood, and other arthropods that scavenge on army ant refuse deposits. The latter have not received much attention, and the few published studies lack detailed species identifications. Here we provide a first systematic inventory of the beetle fauna associated with refuse deposits of *Eciton* army ants, with a focus on *Eciton burchellii*. We collected 8364 adult beetles, 511 larvae, and 24 eggs from 34 deposits at La Selva Biological Station, Costa Rica. We used a combination of DNA barcoding and morphology to identify a subset of 436 specimens to species level. The samples included several new species, and we here formally describe two water scavenger beetles (Hydrophilidae). Refuse deposits harbored a diverse beetle fauna. The identified subset consisted of 91 beetle species from 12 families, with rove beetles being the most abundant and diverse visitors. Of the 85 species found with *E. burchellii*, 50 species were collected from only one or two refuse deposits. Conversely, seven species were found in 10 or more refuse deposits, indicating a certain level of habitat specialization. We matched adults and immatures for 22 beetle species via DNA barcodes, demonstrating that army ant middens also serve as a beetle nursery. The present survey highlights the significant ecological function of army ants as promoters of biodiversity and their status as keystone species in tropical rainforests.

## Graphical abstract

We used DNA barcoding to examine the beetle fauna associated with army ant middens. Middens served as a food source, a breeding ground and a nursery to a diverse assemblage of beetle scavengers. Our study highlights the key role of army ants as promoters of biodiversity.

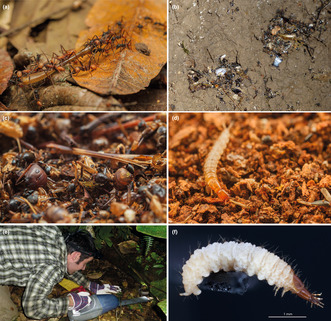

## 1 INTRODUCTION

Scavenging on cadavers is a common foraging strategy in the animal kingdom (Carter et al., 2007; DeVault et al., 2003; Holway & Cameron, 2021; Wilson & Wolkovich, 2011). A famous textbook example are vultures that scavenge on large vertebrate carrion (O'Neal Campbell, 2015). Less conspicuous, but ecologically important, are the omnipresent scavengers of the insect world (Barton et al., 2013; Barton & Bump, 2019; Carter et al., 2007; Holway & Cameron, 2021). These have been intensively studied as consumers of dead vertebrate carcasses including human corpses (Catts & Goff, 1992; Forbes & Carter, 2015; Scott, 1998). However, insects also scavenge on invertebrate corpses (Holway & Cameron, 2021; Mansfield & Hagler, 2016). In fact, invertebrate biomass exceeds that of vertebrates in most ecosystems, making scavenging on invertebrates an ecologically indispensable yet understudied behavior (Barton et al., 2013).

**FIGURE 1:**
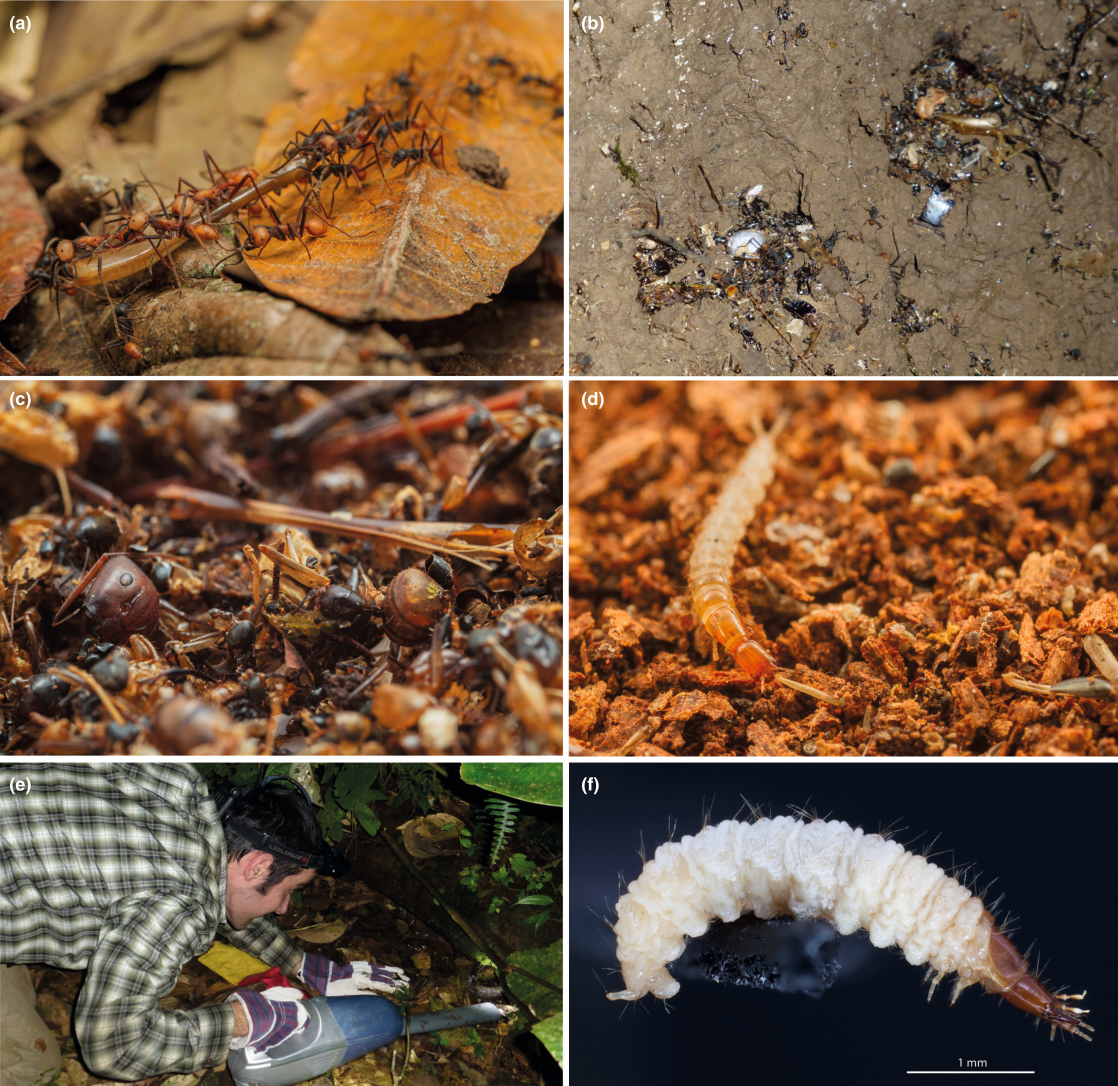
*Eciton burchellii* refuse deposits. (a) Army ant workers cooperatively transport a katydid leg to the colony's bivouac site. (b) Overview of prey remains at a refuse site. The picture only shows a part of the refuse deposit. (c) Close up of arthropod prey remains at a refuse site. (d) Rove beetle larva scavenging on arthropod prey remains in refuse deposit. (e) Collection of refuse deposit with a handheld vacuum cleaner. (f) Focus‐stacked image of a histerid larva found in the deposits (*Clientister* CVB95; sample ID: cvb486hila001).

In 1961, Carl Rettenmeyer described a scavenger hotspot of arthropods in Neotropical rainforests: the refuse deposits of army ants (Rettenmeyer, 1961). Army ant refuse deposits are packed with prey remains. For instance, the army ant *Eciton burchellii* (Westwood, 1842) captures diverse arthropods and can haul in thousands of prey items during a single raid (Franks, 1985; Gotwald Jr, 1995; Hoenle et al., 2019; Powell & Franks, 2006; Rettenmeyer et al., 1983). The prey is generally killed on the spot and, if necessary, dismembered into transportable pieces (Franks, 1985; Gotwald Jr, 1995; Kronauer, 2020; Rettenmeyer, 1963; Schneirla, 1971). The ants carry these prey pieces to the bivouac, the ants' temporary nest (Figure 1a; Gotwald Jr, 1995; Kronauer, 2020). Prey is then fed to the army ant larvae, but leftovers often still contain flesh when dumped at the refuse site (Rettenmeyer et al., 2011). Because of the ants' huge raiding parties, their refuse deposits can contain thousands of dismembered arthropod body parts, representing a nutritionally rich resource for other species (Figure 1b,c; Rettenmeyer, 1961). This accumulation of nutrient‐rich arthropod fragments attracts a variety of visitors such as other ants, mites, springtails, flies, and beetles (Figure 1d; Gotwald Jr, 1995; Kronauer, 2020; Rettenmeyer et al., 2011).

Rettenmeyer (1961) realized that it would take many years to identify and describe this diversity. Many species inevitably remained unidentified in his dissertation and were listed under their genus, tribal or family names (Rettenmeyer, 1961). Even more than 60 years after Rettenmeyer's pioneering work, the refuse‐inhabiting arthropods remain mostly unidentified (Rettenmeyer et al., 2011). This includes the multitude of larvae found in the deposits (Rettenmeyer, 1961; Rettenmeyer et al., 2011). With the advent of molecular techniques, DNA barcoding became available to help detect species boundaries in taxonomically challenging groups (Hajibabaei et al., 2006; Hebert et al., 2004; Janzen et al., 2017) and to match adult and immature stages of the same species (Ahrens et al., 2007; Valdez‐Moreno et al., 2010; Victor et al., 2009). Using this technique, Caterino and Tishechkin (2006) were the first to match a refuse larva to an adult histerid beetle that travels in army ant colony emigrations. This not only established feasibility of this approach, but also suggested that the refuse deposits might be key to the elusive life cycles of obligate army ant guests.

Here, we applied DNA barcoding to inventory the diversity of beetles associated with army ant refuse deposits at a single site—La Selva Biological Station (LSBS), Costa Rica. We focused on 30 deposits of *E. burchellii*, and also inspected three deposits of *Eciton hamatum* (Fabricius, 1782) and one of *Eciton dulcium* Forel, 1912. We established species boundaries based on molecular taxonomic units (MOTUs) (Barcode Index Numbers—BINs) and, in numerous cases, identified the beetles via morphological characters. Like Caterino and Tishechkin (2006), we matched DNA barcodes of different developmental stages, providing basic information on the life history of several refuse‐visiting beetles. We further analyzed the abundance and prevalence of beetles in army ant refuse deposits and provide formal species descriptions for two common water scavenger beetle species. Lastly, we compared the beetle fauna associated with refuse deposits to that associated with army ant colony emigrations.

## 2 METHODS

### 2.1 Collection protocol and research permits

The study took place at LSBS, situated in a lowland Costa Rican tropical rainforest (GPS data: 10°25′19.2″ N, 84°0′54″ W; 35–137 m a.s.l.), during three field seasons between the years 2013 and 2015 (Appendix S1—Specimen information). We collected primarily during the night, when beetle density in refuse deposits has been reported to be higher (Rettenmeyer, 1961). We collected army ant refuse deposits, which consisted of army ant prey remains and the living arthropods therein. The diet of *E. burchellii* consists of a variety of adult arthropods, and prey fragments are transported to the deposits, resulting in comparably large deposits. Because *E. burchellii* nests above ground, these deposits are easily accessible (Rettenmeyer, 1961). In total, 30 spatially separated refuse deposits of 13 *E. burchellii* (subspecies *foreli* Mayr, 1886) colonies were analyzed (Appendix S1—Specimen information). Spatially separated refuse deposits were located at different bivouacking sites of the nomadic army ants. As colony emigrations usually cover distances of around 40–130 m in *E. burchellii* (Franks & Fletcher, 1983; Kronauer et al., 2007; Willis, 1967), the direct overland distances between successive bivouacking sites, and thus refuse deposits, are usually on the order of tens of meters.

Additionally, we collected three refuse deposits of three *E. hamatum* colonies, which also nest above ground but have rather small refuse deposits compared to *E. burchellii*, reflecting their diet of primarily soft‐bodied pupae and larvae of other social insects (Hoenle et al., 2019; Powell, 2011; Powell & Franks, 2006). Furthermore, a hole in the ground covered by a fallen tree branch allowed us to access a small refuse deposit of *E. dulcium*. This species builds its bivouacs below ground (Rettenmeyer, 1963). As refuse deposits are usually located close to, and often directly under the army ant bivouac (Rettenmeyer, 1963; Rettenmeyer et al., 2011), access to refuse sites is generally prevented in below‐ground bivouacking species.

We searched for army ant bivouacs by backtracking ant trails during raids and emigrations, mostly between 8 p.m. and 3 a.m., by walking the trails at LSBS, covering a total area of approx. 11 km^2^. Army ant refuse consisted mainly of arthropod corpse fragments, sometimes also containing empty army ant pupal cocoons in colonies during the late statary phase. We collected middens using a handheld vacuum cleaner with a 30 cm extension tube (Grundig VCH 8830; Figure 1e). The vacuum cleaner contained a fine‐pored filter so that solid material from refuse deposits accumulated in the collection container. After switching off the vacuum cleaner, we immediately transferred the material to plastic boxes that we closed with a rubber lid to prevent flying insects from escaping.

We also collected 15 negative control samples of the forest floor surface where no army ant refuse was present to verify that beetles indeed accumulate in deposits. For this, we searched for spots that looked like typical bivouacking sites of *E. burchellii*, such as cavities under fallen trees or branches, or cavities in the base of trees such as those between buttress roots of tree giants (Kronauer, 2020). We applied the same sampling method as described above and collected an area of approx. 40 cm × 40 cm, which roughly corresponded to the size of larger refuse deposits. For these controls, we only counted the number of individuals without trying to identify the specimens, except for the three histerid specimens detected in the samples (see below).

We sorted the refuse in the field laboratory within 0–4 days after collection (median = 1 day; *N* = 30 *E. burchellii* deposits, *N* = 3 *E. hamatum* deposits, and *N* = 1 *E. dulcium* deposit). We opened the rubber lid several times per day to provide fresh air to the living refuse fauna. Before processing refuse samples, we added a crumbled piece of paper soaked with hexane to the boxes for 10–20 s to anesthetize the insects. Anesthetized specimens were twitching, allowing us to distinguish living material in refuse deposits from dead material. We then stored all specimens of a given refuse in 1.5 mL vials containing absolute ethanol. The present study focuses on adult and juvenile beetles for reasons of feasibility. Other taxa (mostly mites and flies) are deposited at the Technical University of Darmstadt Insect Collection (TUDIC) in absolute ethanol at −30°C for future work.

Colonies of *E. burchellii* undergo stereotypical cycles in which they alternate between a statary and a nomadic phase, which last about 3 and 2 weeks, respectively (Gotwald Jr, 1995; Kronauer, 2020; Schneirla, 1971). The colony stays at the same bivouac site during the statary phase and emigrates to a new one almost every night during the nomadic phase (Gotwald Jr, 1995; Kronauer, 2020). We collected refuse deposits from *E. burchellii* colonies in both phases of the colony cycle: 23 spatially separated refuse deposits of nomadic colonies were sampled once, while seven spatially separated refuse deposits of statary colonies were sampled a total of 14 times (range: 1–4 collections per deposit; Appendix S1—Beetle abundance). The refuse deposits of *E. hamatum* and *E. dulcium* were collected once from colonies in the nomadic phase. Refuse was collected either when colonies were still at the bivouacking site, or up to 2 days after the ants had left.

Collection permits, export permits, and research permits were issued by the ‘Ministry of the Environment, Energy and Technology’ and the ‘National Commission for Biodiversity Management’ (MINAET; permit numbers: 192‐2012‐SINAC, R‐009‐2014‐OT‐CONAGEBIO, and R‐007‐2017‐OT‐CONAGEBIO).

### 2.2 Deposition of specimens, images, and DNA extracts

We deposited 123 specimens of 36 species at eight museum collections (Appendix S1—Specimen information). The remaining material as well as DNA extracts were stored at the TUDIC for future research. Specimen information can be accessed via the Barcode of Life Data (BOLD) Systems website (www.boldsystems.org), which we will update in cases of name and/or depository changes. Additionally, we uploaded 703 focus‐stacked voucher images with scale bars of 285 specimens to BOLD Systems (e.g., Figure 1f and Figure S1 in Appendix S2—Species identification/description; see also Appendix S1—Specimen information). Imaging setups and procedures were described previously (von Beeren, Blüthgen, et al., 2021). Images of adults were mostly taken after DNA extraction, while most larvae were imaged before DNA extraction because larvae often lost their original shape after protein lysis. We have also uploaded high‐resolution images of Figures S1 and S2 to Zenodo (doi:10.5281/zenodo.8199007) to facilitate future taxonomic work.

### 2.3 Species identification and genetic protocol

In total, we collected 8364 adult beetles, 511 beetle larvae, and 24 arthropod eggs from refuse deposits of the three *Eciton* species. No beetle pupae were detected in the refuse. The larvae were identified as belonging to Coleoptera when they had a distinct head with biting mandibles, six segmented thoracic legs, no fleshy prolegs at the abdomen as in caterpillars, and a typical overall habit of beetle larvae. Eggs could not be safely classified as Coleoptera. However, all genetically analyzed larvae and eggs were beetles.

We used specimens from 33 of the 34 collected refuse deposits for DNA barcoding (29 *E. burchellii*, three *E. hamatum*, and one *E. dulcium* deposits; Appendix S1—Specimen information). Usually, we consider DNA barcodes as just one among other informative characters in distinguishing species (Schlick‐Steiner et al., 2010; Tishechkin et al., 2017; von Beeren et al., 2016a, 2016b). However, in the present work, we relied heavily on DNA barcodes to distinguish candidate species for two reasons: First, many of the detected refuse visitors were challenging to identify based on their morphology alone. Second, larval specimens and eggs could only be identified and matched with adult species via DNA barcodes. Detailed morphological studies to name and distinguish the various beetles were beyond the scope of the present work, which focused on providing a first systematic inventory of midden visitors. Nonetheless, we provide formal species descriptions of two new water scavenger beetles and, for a few species, information on diagnostic morphological characters (Appendix S2—Species identification/description).

For a single beetle family, the Histeridae (clown beetles), we identified all specimens found in refuse deposits, and used all of them for DNA barcoding (Appendix S1—Specimen information). We were also able to reliably recognize adults of eight species from additional families based on morphology alone: aff. *Pridonius* CVB70, *Cephaloplectus mus*, *Cercyon pohli* sp. nov., *Ecitochara connexa*, *Ecitomorpha* cf. *nevermanni*, *Ecitomorpha* cf. *breviceps*, *Ecitophya simulans*, and *Sacosternum laselva* sp. nov. Note that not all the specimens of these species were used for DNA barcoding. The abundance (see section ‘Beetle abundance and prevalence’) of non‐barcoded but identified specimens is given in Appendix S1—Beetle abundance. The prevalence of these eight species and of all histerid beetles are expected to best represent the actual prevalence in army ant refuse deposits, although we might have missed to analyze the larvae of these species in some of the deposits. The prevalence of many other species was most likely underestimated in the present work, because we only analyzed a relatively small proportion of the entire refuse beetle samples via DNA barcoding.

We aimed to acquire DNA barcodes for a subset of 548 beetle specimens (376 adults, 165 larvae, seven eggs). The number of refuse visitors used for genetic analysis ranged from a minimum of two specimens to a maximum of 62 specimens per deposit (mean ± SD = 17 ± 13; median = 14.5; Appendix S1—Specimen information). For each deposit, we chose to analyze adults and larvae that looked morphologically distinct to cover a broad taxonomic spectrum, but we likely missed some species in most deposits. Our screening of refuse visitors is thus far from complete, considering that we collected many thousand beetles, particularly of the diverse rove beetle subfamily Aleocharinae.

We extracted DNA using QIAGEN DNeasy Tissue Kits for single extractions and for 96‐well plates. We followed the standard protocol but did not homogenize specimens to keep specimens intact for morphological studies (von Beeren et al., 2016b; von Beeren, Blüthgen, et al., 2021). All DNA extracts were stored at −30°C at the TU Darmstadt to serve as DNA vouchers. We amplified the classical animal DNA barcode region of the mitochondrial gene *cytochrome oxidase I* (*COI*) in polymerase chain reactions (PCRs). PCRs were set up as described previously (von Beeren et al., 2016a, 2016b). We used various published and custom PCR primers for DNA amplification (see Appendix S2—PCR primer combinations used in this study). Purification and sequencing of PCR products were outsourced to Macrogen USA (New York City, USA; 2013–2015) and to Macrogen Europe (Amsterdam, The Netherlands; 2016–2023). PCR amplicons were always sequenced in forward and reverse directions using Sanger sequencing. In cases of low‐quality reads, PCR settings were adjusted and sequencing was repeated.

We used the software Geneious Prime (version 2022.1.1; https://www.geneious.com) for sequence analyses, including assembly of forward and reverse sequences, sequence trimming, sequence editing, sequence alignment using the MUSCLE algorithm (Edgar, 2004), and clustering analyses. We performed several quality checks with the resulting consensus sequences. Sequences with gaps or stop codons in the *COI* alignment were sorted out as they likely represented nuclear mitochondrial pseudogenes (*N* = 15 *COI* sequences). We compared barcoding results to morphological identifications to detect and omit apparently erroneous sequences due to contamination or pipetting errors (<1% of DNA barcodes). Final consensus sequences were deposited at the BOLD Systems. GenBank accession numbers are given in Appendix S1—Specimen information.

Using the clustering algorithm RESL, BOLD Systems assign “BINs” to each uploaded specimen that is associated with a DNA barcode. BINs define distinct genetic units in the entire BOLD Systems database (Ratnasingham & Hebert, 2013). We used these BINs to identify MOTUs in our DNA barcode dataset. A main advantage of BINs is that they provide a standardized analytical tool to identify MOTUs, without each study defining their own sequence similarity thresholds (for a critical discussion see Sharkey et al., 2021; Zamani et al., 2022). To assess the robustness of sequence partitioning, we employed a second clustering method known as “Assemble Species by Automatic Partitioning” (ASAP; Puillandre et al., 2021). ASAP is designed to deduce species partitions from single locus sequence alignments like DNA barcode data. It utilizes a hierarchical clustering algorithm that relies on pairwise genetic distances. For our dataset, we employed simple p‐distances. The algorithm identifies the 10 most promising partitions and assigns an ASAP score to each of them. A lower ASAP score indicates a better partition quality. In contrast to the BINs generated in BOLD Systems, it allows inclusion of sequences shorter than 300 bp.

We identified beetle taxa based on DNA barcode similarity to the reference barcodes in BOLD Systems. We used species names when a sequence was clustered within an existing BIN with a species name (see above), genus names when a sequence match was between ≥95% and <99%, and family names when sequences matched between ≥90% and <95%. In cases where a sequence match was ≥80% and <90%, we adopted the order name Coleoptera. Some sequences were not clustered into a BIN because BOLD Systems will not create a new BIN for sequences ≥300 and <500 bp and sequences <300 bp are not considered in the BIN analyses (Ratnasingham & Hebert, 2013).

For adult beetles, we verified BIN identifications using morphological characters. We adopted our morphological identifications, if these allowed us to provide lower taxonomic units than achieved via DNA barcode similarity. The column titled “Identification approach” in “Appendix S1—Specimen information” provides information on whether morphological identification superseded the identification based on DNA barcode similarity. For beetles that were not identified to species level, the species names provided in the present work represent the lowest taxonomic level we were able to identify, plus a unique identifier (e.g., genus level: *Ecitodonia* CVB65). For nine previously studied species (von Beeren, Blüthgen, et al., 2021), we adapted the previous species naming as follows (old name from von Beeren, Blüthgen, et al., 2021/new name): *Ecitodonia* sp. 1/*Ecitodonia* CVB65; *Ecitopora* sp. 2/*Ecitopora* CVB63; *Euclasea* sp. 1/*Euclasea* CVB66; False‐Lomechusini sp. 1/False‐Lomechusini CVB67; False‐Lomechusini sp. 2/False‐Lomechusini CVB68; *Limulodes* sp. 3/*Limulodes* CVB64; *Myrmedonota* sp. 2/*Myrmedonota* CVB69; *Quedius* (*Pridonius*) sp. 1/aff. *Pridonius* CVB70; *Vatesus* cf. *clypeatus* sp. 2/*Vatesus* cf. *clypeatus* CVB72.

For visual representation of genetic data, we generated neighbor‐joining (NJ) trees based on Tamura‐Nei distances using the Geneious Tree Builder. For better visualization, NJ trees were rooted using a *COI* sequence of *Hydroscapha takahashii* Miwa, 1935 (GenBank accession number: MT132896.1), a beetle of the suborder Myxophaga (family Hydroscaphidae; Fikáček et al., 2020). We used NJ trees because our primary focus was on species identification and candidate species delineation based on genetic distances. In addition, we provide a RAxML tree in Appendix S3, which includes full terminal labels for reference, allowing for further exploration of potential phylogenetic relationships within the dataset.

### 2.4 Beetle abundance and prevalence

We restricted our analyses of beetle abundance and prevalence to the species found in *E. burchellii* deposits, because refuse sample sizes of other *Eciton* species were too small to draw robust conclusions. We analyzed the beetle abundance and prevalence data in R (version 4.2.2). The abundance of beetles (number of beetle specimens) collected from *E. burchellii* refuse deposits in the statary phase (statary refuse), in the nomadic phase (nomadic refuse), and from control plots were compared using the Kruskal–Wallis test (Kruskal & Wallis, 1952). As post hoc analysis we used Dunn's multiple comparisons rank sum test (Dunn, 1964) with false discovery rate correction (Benjamini & Hochberg, 1995) to account for Type I error accumulation (R package PMCMRplus; Pohlert, 2018; Pohlert & Pohlert, 2018). To ensure comparability of the data from statary refuse deposits that we resampled on different days, we used exclusively the abundance data from the first collection event in the analysis (Appendix S1—Beetle abundance).

Besides abundance, we analyzed the beetle prevalence in *E. burchellii* refuse deposits to distinguish sporadic from regular visitors of army ant middens. We defined “prevalence” of a beetle species as the number of different (spatially separated) refuse deposits that contained at least one specimen of that species (including adults and immatures). This includes sampling of spatially separated refuse deposits at different bivouacking sites of the same colony. If the same species was recollected on different days from the same statary deposit (Appendix S1—Beetle prevalence), we counted it only once.

We used the prevalence data of refuse‐visiting species to estimate the adequacy of our refuse deposit survey in representing the actual beetle fauna by running species accumulation curves using the R function specaccum() incorporated in the package vegan (Oksanen et al., 2022). We assigned the 30 analyzed *E. burchellii* refuse deposits randomly (method = “random”) and ran 1000 permutations. Additionally, we used the function specpool() to predict the extrapolated species richness. We used the R package ggplot2 (Villanueva & Chen, 2019) for plotting a histogram showing the number of times a given species was present in spatially separated *E. burchellii* refuse deposits.

The abundance, prevalence, and species richness of beetles in army ant refuse deposits may be influenced by various factors not addressed in this study, such as refuse size/volume or the specific collection site. Unfortunately, we did not measure the size of the deposits or collect spatial coordinates and were therefore not able to account for these variables in our statistical analysis. However, the main goal of this study was not to test ecological drivers of diversity, such as differences in habitat or refuse volume, but to compile an initial inventory of beetle diversity at refuse sites.

### 2.5 Spatial niche differentiation between microhabitats

Army ants are nomadic and emigrate to new bivouac sites on a regular basis (Gotwald Jr, 1995; Kronauer, 2020; Schneirla, 1971). Many of the more closely‐associated, obligate army ant guests participate in these emigrations either on foot, or as hitchhikers that are carried by the ants (Gotwald Jr, 1995; Kronauer, 2020; von Beeren, Blüthgen, et al., 2021; von Beeren & Tishechkin, 2017). By using various integration mechanisms such as chemical host mimicry or protective morphologies, these guests often live within the army ant bivouacs among the host workers (Gotwald Jr, 1995; von Beeren et al., 2018; von Beeren, Blüthgen, et al., 2021; von Beeren, Brückner, et al., 2021). Refuse‐visiting species were described as mostly distinct from those following the ants' colony emigrations (Akre & Rettenmeyer, 1966; Gotwald Jr, 1995; Rettenmeyer, 1961). However, this separation has not been quantified. We therefore assessed the degree of spatial niche differentiation in *E. burchellii* associates at LSBS using a bipartite interaction network (e.g., Ivens et al., 2016). We used the function “visweb()” as implemented in the R package ‘bipartite’ (Dormann et al., 2009) to visualize the prevalence of beetle species in refuse deposits and emigrations. Importantly, we defined the prevalence differently for emigrations and refuse deposits. The prevalence data of beetles in emigrations were taken from von Beeren, Blüthgen, et al. (2021). Here, we sometimes recollected from emigrations of the same colony (*N* = 13 colonies; *N* = 27 emigrations; von Beeren, Blüthgen, et al., 2021), but only counted each beetle species once for any given colony. Accordingly, the maximum possible value for prevalence during *E. burchellii* colony emigrations was 13. We consider recollections of emigrations of the same colony on different days as highly dependent collection events. By collecting beetles from emigrations, we certainly decreased the chances of collecting the same beetle species again during consecutive emigration collections of the same colony. Hence, we decided to use the presence of beetles in different colonies as the prevalence for emigration‐following beetles (Appendix S1—Network; von Beeren, Blüthgen, et al., 2021). In contrast, we consider different refuse deposits as spatiotemporally independent collection events irrespective of colony affiliation. This is because most of the refuse‐visiting beetles do not follow emigrations and thus refuse deposits are expected to be recolonized each time, irrespective of which colony produced the deposit. We are aware, however, that the occurrence of a couple of beetle species in deposits might not be entirely independent of colony identity as some emigration followers were also found in the deposits (see below). We thus also provide data on colony‐level prevalence of refuse visitors in Appendix S1—Network, which mostly mirrors the pattern of niche differentiation from the analysis at the level of spatially separated refuse deposits.

## 3 RESULTS

### 3.1 Beetle abundance at army ant refuse sites

**FIGURE 2:**
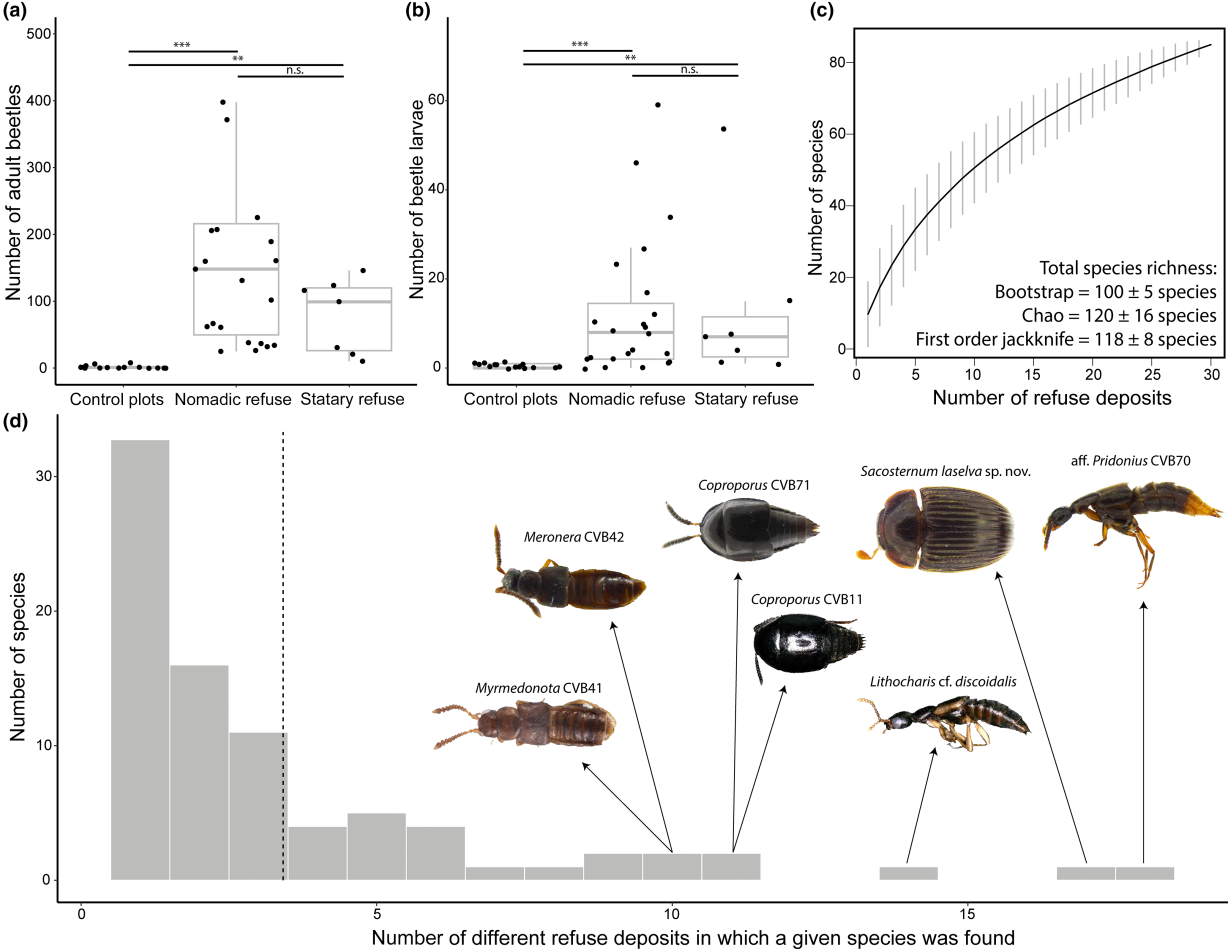
Abundance, species accumulation curve, and prevalence of beetles in *Eciton burchellii* refuse deposits. Number of (a) adult beetles and (b) beetle larvae in control plots, refuse deposits of colonies in the nomadic phase, and refuse deposits of colonies in the statary phase. For the four resampled statary refuse deposits, we only considered the first refuse collection in the comparison to nomadic refuse deposits, which were sampled only once. Boxplots show median and inter‐quartile range; whiskers extend from the hinge to the highest/lowest value that is within 1.5 times of the inter‐quartile range. In (a), three outliers are not shown for improved clarity (685 specimens, 1016 specimens, and 2705 specimens in nomadic refuse). (c) Species accumulation curve (black line) with standard deviation (gray bars). The extrapolated species richness, that is, the predicted number of actual refuse visitors in the population, is given using three different estimation models (mean ± SD). (d) Histogram showing the prevalence of beetle species in 30 spatially separated refuse deposits of *E. burchellii* at La Selva Biological Station. Scale bars for all specimen images are provided in the files deposited at BOLD Systems. Dashed line shows the mean. ***p < .001, **p < .010, ^n.s.^ *p* ≥ .050, Dunn's post hoc test.

We collected a total of 8014 adult beetles (mean ± SD: 267 ± 506 adults per refuse, median = 140), 504 beetle larvae (mean ± SD: 17 ± 22 larvae per refuse, median = 7.5), and 18 eggs (mean ± SD: 1 ± 1 eggs per refuse, median = 0) from 30 spatially separated *E. burchellii* refuse deposits (Appendix S1—Beetle abundance). Beetle abundance was highly variable (Figure 2a,b). The number of adult beetles per refuse deposit ranged from a minimum of 10 to a maximum of 2705 specimens. With 7038 adults, rove beetles (Staphylinidae) were by far the most abundant beetles in *E. burchellii* refuse deposits, followed by feather‐winged beetles (Ptiliidae; *N* = 650 adults), water scavenger beetles (Hydrophilidae; *N* = 119 adults), round fungus beetles (Leiodidae; *N* = 65 adults), and clown beetles (Histeridae; *N* = 46 adults).

Beetle abundance differed between nomadic deposits, statary deposits, and control plots (Kruskal–Wallis test, adults: *χ* ^2^ = 31.11, df = 2, *p* < .001; larvae: *χ* ^2^ = 18.95, df = 2, *p* < .001; Figure 2a,b). Irrespective of the colony's phase, army ant refuse deposits yielded higher numbers of both adult and larval beetles, compared to control plots (Dunn's post hoc test, *p* ≤ .007; Figure 2a,b). However, there was no discernible difference in the number of adult or larval beetles in refuse deposits from the statary phase compared to the nomadic phase (Dunn's post hoc test, adults: *p* = .205, larvae: *p* = .895; Figure 2a,b). Noteworthy, we collected three specimens of the histerid beetle *Plagiogramma schmidti* (Wenzel & Dybas, 1941) in a single control plot (specimen IDs: cvb417hist020, cvb417hist021, cvb421hist020; deposited at the California State Collection of Arthropods), a species that we also collected in one refuse deposit each of *E. burchellii* and *E. hamatum* (Appendix S1—Specimen information).

We additionally collected 347 adult beetles (mean ± SD: 116 ± 100 adults per refuse), as well as six larvae (mean ± SD: 2 ± 2 larvae per refuse) and six eggs (mean ± SD: 2 ± 2 eggs per refuse) from three *E. hamatum* deposits (Appendix S1—Beetle abundance). Only three adults and one larva were recovered from the single *E. dulcium* deposit.

### 3.2 Species identification and species richness

**FIGURE 3:**
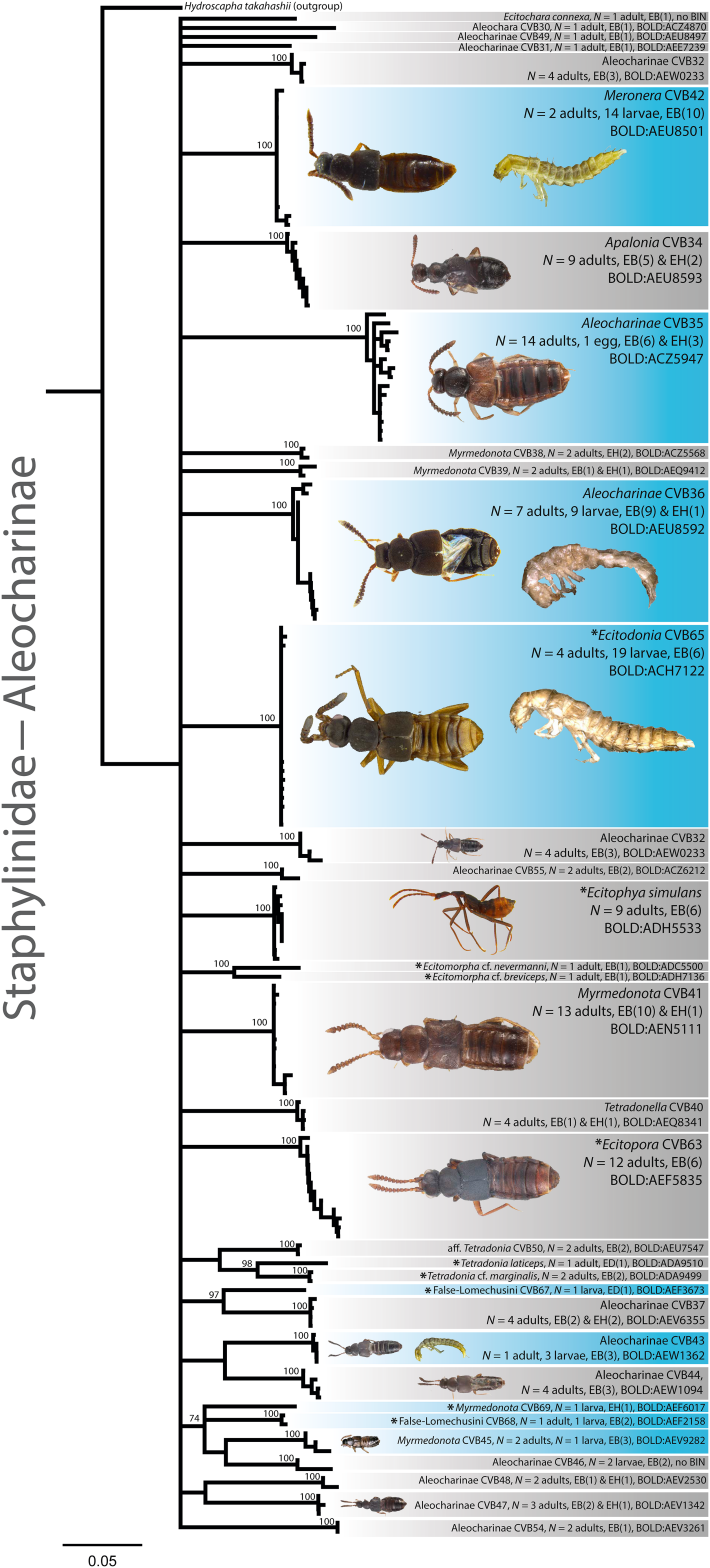
Clustering of DNA barcode data of aleocharine rove beetles. Neighbor‐joining tree based on beetle *COI* sequences to visualize genetic distances. Species delimitations are based on BINs, which are provided for each species (e.g., *Ecitopora* CVB63, BIN = BOLD:AEF5835). Light‐blue boxes highlight species with a DNA barcode match between adults and immatures. For the two aleocharine species False‐Lomechusini CVB67 and *Myrmedonota* CVB69, we only detected beetle larvae in the present study but found a DNA barcode match to adults collected previously from *Eciton* emigrations (von Beeren, Blüthgen, et al., 2021). Gray boxes mark species where only adults or only immatures were detected. Asterisks mark species that were also found in *Eciton* emigrations (von Beeren, Blüthgen, et al., 2021). The army ant species from which a given refuse visitor was collected is given by the abbreviations EB (*Eciton burchellii*), EH (*Eciton hamatum*), and ED (*Eciton dulcium*). The number of times a species was detected in spatially separated refuse deposits is given in parentheses. Note that we additionally identified specimens without DNA barcodes. The prevalence can thus be higher than the number of barcoded specimens. Bootstrap support values are provided at terminal nodes. Nodes with bootstrap support ≤50 were collapsed. The scale bar indicates Tamura‐Nei distances. *Hydroscapha takahashii* (GenBank accession number: MT132896; Fikáček et al., 2020), a beetle of the suborder *Myxophaga*, served as the outgroup. Scale bars for all specimen images are provided in the files deposited at BOLD Systems.

**FIGURE 4:**
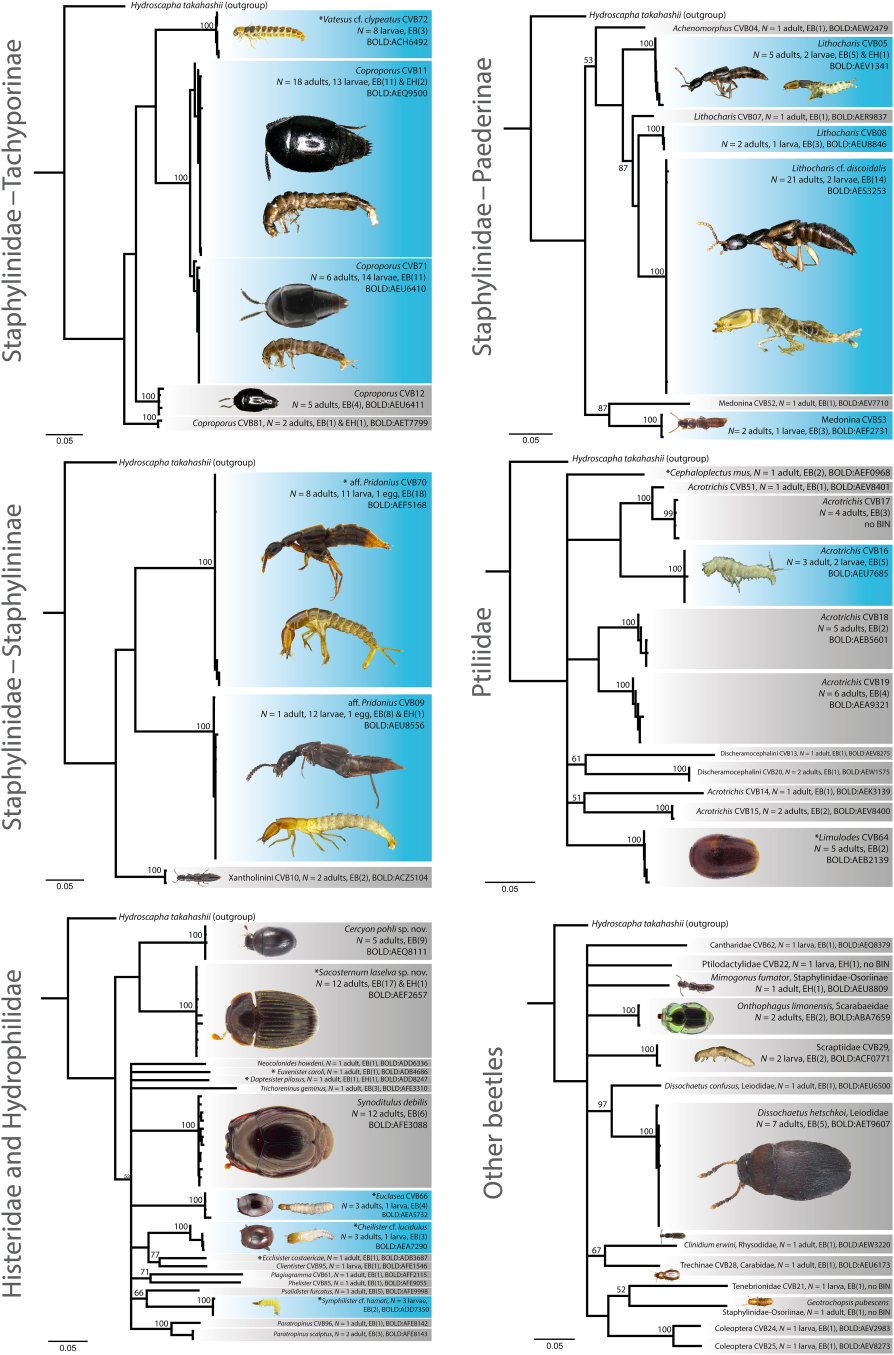
Clustering of DNA barcode data of remaining beetles. Neighbor‐joining trees based on beetle *COI* sequences. Colors of boxes and abbreviations correspond to Figure 3. For the tachyporine *Vatesus* cf. *clypeatus* CVB72 and the histerid *Symphilister* cf. *hamati*, we only detected larvae in the present study but found DNA barcode matches to adults collected previously from *Eciton* emigrations (von Beeren, Blüthgen, et al., 2021). Note that we identified additional specimens without DNA barcodes. The prevalence can thus be higher than the number of barcoded specimens (e.g., *Cercyon pohli* sp. nov.). Bootstrap support values are provided at terminal nodes. Nodes with bootstrap support ≤50 were collapsed. The scale bars indicate Tamura‐Nei distances. *Hydroscapha takahashii* (GenBank accession number: MT132896; Fikáček et al., 2020), a beetle of the suborder *Myxophaga*, served as the outgroup. Scale bars for all specimen images are provided in the files deposited at BOLD Systems.

We successfully DNA barcoded 414 specimens (281 adults, 130 larvae, and 3 eggs; Figures 3 and 4), while PCR amplification repeatedly failed for 134 specimens (95 adults, 35 larvae, and 4 eggs; Appendix S1—Specimen information). Despite missing DNA barcodes, we were able to identify 22 of the latter specimens solely based on their morphology. In total, we thus identified 436 beetle specimens, which include specimens identified to the species level (e.g., *Ecitophya simulans*) and those that were defined by DNA barcode similarity but missed an identification to the species level (e.g., Cantharidae CVB62).

The *COI* sequences were assigned to 84 BINs by the BOLD Systems clustering approach (Appendix S1—Specimen information). These BINs were used as a proxy for species in the present study (see Figures 3 and 4; Appendix S1—Specimen information). In addition to the 84 species assigned via BINs, we assigned six species for specimens with distinct DNA barcodes that were too short in length to be considered in the BIN analysis (see next paragraph). The best scoring species partitioning detected by the clustering algorithm ASAP also detected 90 genetic candidate species (ASAP score: 1.00; Appendix S1—ASAP partitioning). Most of the candidate species recognized by ASAP were the same as those identified by the BIN approach, with three exceptions: *Coproporus* CVB71 was divided into three candidate species, while the species pairs *Acrotrichius* CVB17 and *Acrotrichius* CVB51, as well as *Lithocharis* CVB07 and *Lithocharis* CVB08, were each grouped into a single candidate species (Appendix S1—ASAP partitioning).

Seventeen *COI* sequences were not assigned to a BIN, because sequences were too short (range = 156–432 bp, median = 303 bp). Seven of these showed between 99.41% and 100% sequence similarity to adult beetles that were assigned to a BIN, and the specimens were thus assigned to the same species (one specimen of *Coproporus* CVB11, three specimens of *Sacosternum laselva* sp. nov., two specimens of aff. *Pridonius* CVB70, and one specimen of *Meronera* CVB42; Appendix S1—Specimen information). The remaining 10 specimens represented six additional species that we identified as follows: The *COI* sequences of two larvae (sample IDs: cvb459_l1 & cvb296_l2) matched to the following records in the BOLD Systems database: the former showed a 99.22% match to a beetle that was identified as a member of the family Ptilodactylidae (Sequence ID: PLBAD023‐18.COI‐5P), and the latter showed a 97.60% match to a beetle that was identified as a member of the family Tenebrionidae (unpublished record). These two larvae were thus assigned family‐level IDs. Further, four adults were morphologically identified as ptiliid beetles of the genus *Acrotrichis* (species ID: *Acrotrichis* CVB17), one specimen as *Ecitochara connexa*, one specimen as *Geotrochopsis pubescens*, and two specimens as aleocharine beetles (species ID: Aleocharinae CVB46; Appendices S1—Specimen information and S2—Species identification/description). For a single identified species, we failed to acquire a DNA barcode: The species *P. schmidti* (two specimens in refuse deposits and three specimens in a control plot) was identified solely based on morphological characters (Appendix S2—Species identification/description).

Altogether, we detected 91 beetle species in army ant middens (Figures 3 and 4; Appendix S1—Specimen information). For 53 of them, our morphological identification of adult beetles superseded the identification based on DNA barcode similarity to the references in BOLD Systems, 37 species were solely identified by DNA barcode similarity, and the identification of one species was solely based on morphology (Appendix S1—Specimen information). Several of these species are new to science and have not been taxonomically described (Appendix S2—Species identification/description). When focusing on *E. burchellii* alone, we detected 85 beetle species in its refuse deposits (Figures 3 and 4; Appendix S1—Specimen information). A species accumulation curve as well as estimates of species richness under more intense sampling indicated that the actual species number of refuse scavenging beetles at LSBS is about 1.18–1.41 times higher (Figure 2c).

Of the 91 species found in total, we were able to provide species names for 28, while other species were identified to the level of genus (*N* = 35 species), (sub)tribe (*N* = 7 species), subfamily (*N* = 15 species), family (*N* = 4 species), or (sub)order (*N* = 2 species). Refuse‐associated beetles belonged to the following 12 families: Cantharidae (*N* = 1 species), Carabidae (*N* = 1 species), Histeridae (*N* = 16 species), Hydrophilidae (*N* = 2 species), Leiodidae (*N* = 2 species), Ptiliidae (*N* = 11 species), Ptilodactylidae (*N* = 1 species), Rhysodidae (*N* = 1 species), Scarabaeidae (*N* = 1), Scraptiidae (*N* = 1), Staphylinidae (*N* = 51), and Tenebrionidae (N = 1).

### 3.3 Formal species descriptions

Two common refuse‐visiting water scavenger beetles (family Hydrophilidae) represented undescribed species. Here we provide concise formal taxonomic descriptions for these species. Detailed species descriptions are given in Appendix S2—Species identification/description. To follow the guidelines of zoological nomenclature, the article was registered under the ZooBank LSID number urn:lsid:zoobank.org:pub:AB171F8F‐A44F‐4498‐AD19‐9067D713A1D6. A high‐resolution image of Figure S2 has also been deposited at Zenodo (doi:10.5281/zenodo.8199007).

***Cercyon pohli* Fikáček sp. nov.**

Habitus images and relevant characters are illustrated in Appendix S2—Figure S2a–g. The ZooBank LSID number of the species is urn:lsid:zoobank.org:act:D2DD16C6‐C5B8‐4AA0‐A917‐1EF92E81B62F.

**Type material. Holotype:** 1 male: “COSTA RICA: Heredia Prov., La Selva Biol. Station, 24.ii.2013, 10°25.838205′ N 84°0.418470′ W, refuse deposit of *E. burchellii*, leg. C. v. Beeren & S. Pohl, Colony ID: EB01, sample ID: cvb117roun001, *COI* GenBank accession number: OQ173065,” deposited in the National Museum, Prague, Czech Republic. **Paratypes:** 4 specs., see Appendix S2—Detailed species description.

**Diagnosis.** By the combination of the small body size, dorsal coloration without darker spots, absence of femoral lines, and the form of the aedeagus, *C. pohli* sp. nov. is very similar to *C. integer* Sharp, 1882. We examined *C. integer* specimens from Costa Rica (Guanacaste and Puntarenas provinces) and compared them to types from Mexico and Guatemala. Clearly, *C. integer* differs from *C. pohli* sp. nov. in (1) distinctly impressed elytral series, (2) much smaller eyes with eyes being separated by 6× the width of one eye, (3) wider aedeagus with relatively shorter parameres (1/4× the length of phallobase), and (4) very narrow median portion of male sternite 9. Other small‐sized Central American species without femoral lines can be distinguished from *C. pohli* sp. nov. by larger dark body and an aedeagus with long parameres with a widely expanded apex (*C. ebeninus* Sharp, 1882), or by a pronotum with central dark spots (*C. variegatus*; Arriaga‐Varela et al., 2017).

***Sacosternum laselva* Fikáček sp. nov.**

This species was previously analyzed in a molecular phylogenetic study (as *Sacosternum* sp.; Arriaga‐Varela et al., 2021) and in two studies addressing the host specificity and integration mechanisms of *Eciton* guests (as *Sacosternum* aff. *lebbinorum*; von Beeren, Blüthgen, et al., 2021; von Beeren, Brückner, et al., 2021). Habitus images and relevant character illustrations are provided in Appendix S2—Figure S2h–o. The ZooBank LSID number of the species is urn:lsid:zoobank.org:act:712ACAEC‐0778‐4804‐9506‐E12A21406024.

**Type material. Holotype:** 1 male: “COSTA RICA: Heredia Prov., La Selva Biol. Station, 9.ii.2013, 10°25.847′ N 84°0.404′ W, collected in emigration of *E. burchellii foreli*, leg. C. v. Beeren, colony ID: EB01, sample ID: cvb015miro001, *COI* GenBank accession number: MW129390,” deposited in the National Museum, Prague, Czech Republic. **Paratypes:** 2 specs., see Appendix S2—Detailed species description.

**Diagnosis.** *Sacosternum laselva* sp. nov. is very similar to *S. lebbinorum* Fikáček and Short (2010) in the form of the prosternum (with lateral extensions; Appendix S2—Figure S2k, arrow), the lateral part of the metaventrite (with the triangular area; Appendix S2—Figure S2l, arrow), the form of the mentum (widely pentagonal), the form of the mesoventral plate (narrowly elongate; Appendix S2—Figure S2i,l), and the apex of the median lobe (without lateral sclerites; Appendix S2—Figure S2n,o). It also keys to *S. lebbinorum* in the identification key by Fikáček and Short (2010). It can be distinguished from *S. lebbinorum* by the form of the median lobe (bottle‐shaped, i.e., wide basally and with apical narrow part in *S. laselva* sp. nov., narrow from base to apex in *S. lebbinorum*), the presence of a gonopore (indistinct in *S. lebbinorum*), and the apical sclerite of the median lobe being very narrow at the apex (widely triangular in *S. lebbinorum*).

### 3.4 Species prevalence at refuse sites

Most species showed a low prevalence, that is, they were found in only a few spatially separated refuse deposits of *E. burchellii* (Figure 2d; mean ± SD = 3.39 ± 3.59 different deposits per species; median = 2; *N* = 85 species). Thirty‐four species were only detected in a single *E. burchellii* refuse and 16 species in two deposits (Figures 2, 3, 4). On the other hand, 20 species were found in five or more deposits, and the following seven species were detected in at least 10 deposits: aff. *Pridonius* CVB70 (*N* = 18 deposits), *Coproporus* CVB11 (*N* = 11 deposits), *Coproporus* CVB71 (*N* = 11 deposits), *Lithocharis* cf. *discoidalis* (*N* = 14 deposits), *Meronera* CVB42 (*N* = 10 deposits), *Myrmedonota* CVB41 (*N* = 10 deposits), and *Sacosternum laselva* sp. nov. (*N* = 17 deposits; Figures 2, 3, 4; Appendix S1—Beetle prevalence).

Twenty species were detected in the three *E. hamatum* deposits, and two species were detected in the single *E. dulcium* deposit. Both species found in the *E. dulcium* deposit were unique refuse visitors of this army ant species (False‐Lomechusini CVB67, *Tetradonia laticeps*), while four species were unique to *E. hamatum* deposits (*Myrmedonota* CVB38, *Myrmedonota* CVB69, Ptilodactylidae CVB22, Staphylinidae CVB23). The remaining 16 species were also detected in *E. burchellii* refuse deposits (Figures 2, 3, 4; Appendix S1—Beetle prevalence).

### 3.5 Adult‐immature matches

Of the 91 detected beetle species in *Eciton* refuse deposits, 61 species were represented by adults alone, 12 species by larvae or eggs alone, and for 18 species, we detected both adult and immature stages (Figures 3 and 4; Appendix S1—Life stages). Integrating previously published information on emigration followers of *Eciton* army ants (von Beeren, Blüthgen, et al., 2021), we were able to match adults and immatures of four additional species, resulting in an overall adult–immature match for 22 beetle species (Figures 3 and 4; Appendix S1—Life stages). We found larvae of nine species in refuse deposits that were also collected in army ant colony emigrations: aff. *Pridonius* CVB70, *Cheilister* cf. *lucidulus*, *Ecitodonia* CVB65, *Euclasea* CVB66, False‐Lomechusini CVB67, False‐Lomechusini CVB68, *Myrmedonota* CVB69, and *Symphilister* cf. *hamati* participated in the emigrations as adults, while both larvae and adults of *Vatesus* cf. *clypeatus* CVB72 (Figures 3 and 4) were found during emigrations (Akre & Torgerson, 1969; von Beeren et al., 2016b).

### 3.6 Spatial niche differentiation between microhabitats

**FIGURE 5:**
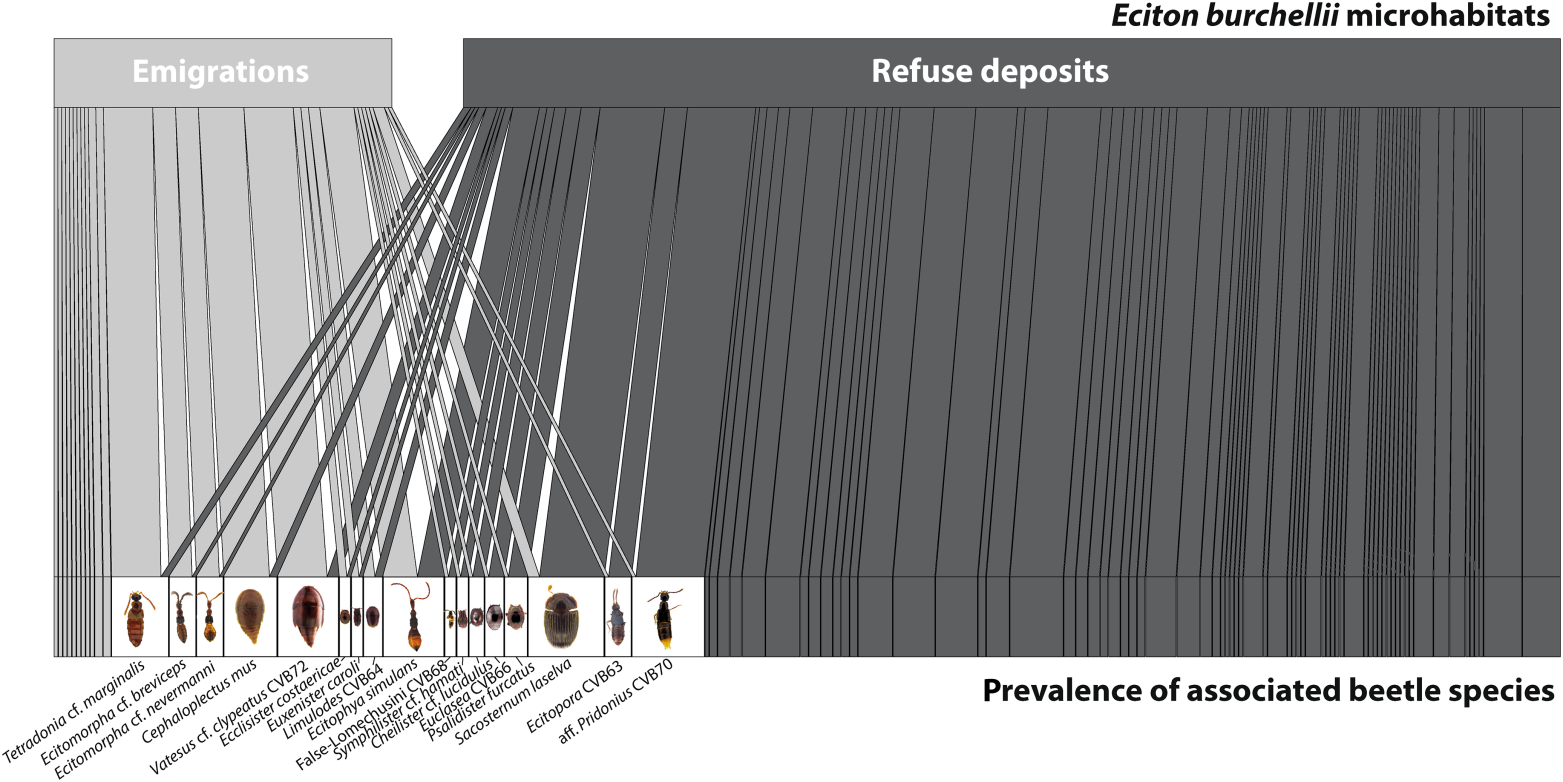
Spatial niche differentiation in beetles associated with *Eciton burchellii*. Bipartite network graph showing the prevalence of 96 beetle species in emigrations and refuse deposits of *E*. *burchellii*. Beetle species are depicted as gray or white boxes, with white boxes highlighting those species occurring in both microhabitats. Note that these species are marked with asterisks in Figures 3 and 4. Beetle prevalence in either emigrations or refuse deposits is depicted by connecting lines. For emigrations, line width is proportional to the number of times a species was detected in different *E. burchellii* colonies (13 analyzed colonies, 27 emigrations; von Beeren, Blüthgen, et al., 2021). For refuse deposits, line width represents the number of times a given beetle was present in spatially separated *E*. *burchellii* refuse deposits (30 analyzed refuse deposits; 13 colonies; Appendix S1—Network). Network links are colored according to the microhabitat. The network is optimized to possess as few crossings of links as possible. The network matrix is given in Appendix S1—Network.

Of the 96 beetle species found in *E. burchellii* refuse deposits and/or emigrations (Appendix S1—Network), 17 species were found in both microhabitats, 68 species were exclusively found in the deposits, and 11 species exclusively in colony emigrations (Figure 5; Appendix S1—Network). Among the beetles found in both microhabitats, it is noticeable that prevalent emigration followers were infrequently found in refuse deposits (e.g., *Cephaloplectus mus*, *Ecitomorpha* cf. *nevermanni*, and *Tetradonia* cf. *marginalis*) and prevalent refuse visitors infrequently in emigrations (e.g., aff. *Pridonius* CVB70, *Ecitopora* CVB63, and *Sacosternum laselva* sp. nov.; Figure 5; Appendix S1—Network). One exception is *Ecitophya simulans*, which was common in both microhabitats (Figure 5).

## 4 DISCUSSION

We found the astonishing number of 2705 beetle specimens in a single army ant midden, highlighting that “refuse deposits are teeming with life”, as aptly put by Rettenmeyer et al. (2011). Building on their pioneering work (Rettenmeyer, 1961; Rettenmeyer et al., 2011), our study aimed to catalog the deposits' diverse beetle fauna. Like our predecessors, we faced obstacles in identifying the beetles, which we overcame by utilizing DNA barcoding. Through this method, we were able to group the diversity into genetic clusters, or BINs, which we used as a proxy for species identity. With over 8000 specimens and 91 species, we discovered an astonishing abundance and diversity of beetles, affirming that army ant middens are a hotspot for arthropod scavengers in tropical rainforests. Based on the species accumulation curve, we believe that the actual number of beetle species in these deposits is likely even higher, and when we consider other taxa such as mites, flies, and springtails, it is reasonable to assume that the diversity of species in army ant middens numbers in the several hundred range in any given population.

Previous studies have viewed refuse‐visiting species as opportunistic leaf litter scavengers and detritivores (Gotwald Jr, 1995; Rettenmeyer, 1961). The collection of the histerid beetle *Plagiogramma schmidti* from one forest floor control sample as well as from two *Eciton* deposits supports this supposition. *Plagiogramma* beetles scavenge on all kinds of fungal spores (Kovarik & Caterino, 2016), which might explain their presence in both microhabitats. All other refuse‐visiting histerids are carnivores (Kovarik & Caterino, 2016) that were likely attracted to the accumulation of prey remains in the deposits. Most of the species we encountered in our survey were rare and only present in one or two deposits, likely representing opportunistic leaf litter scavengers. On the other hand, several species were common and regularly found in *E. burchellii* deposits. It is tempting to speculate that these common visitors are specialized to utilize this microhabitat and actively seek out army ant middens. This could represent an initial evolutionary step toward a greater dependence on army ants. For example, we commonly found paederine rove beetles of the genus *Lithocharis* Dejean, 1833 in the deposits, which are known scavengers on all sorts of decaying organic matter (Assing, 2015). Such species seem to be preadapted to transition from a more opportunistic scavenging lifestyle to a refuse deposit specialist if this switch increases their reproductive fitness. Notably, *Lithocharis* cf. *discoidalis* was found in 14 *E. burchellii* deposits, suggesting that a certain level of habitat specialization might already exist in this species.

Odorants could play an important role as attractants for refuse visitors. The smell of animal carcasses, including those of dead arthropods (Schmitt et al., 2004), is known to attract a variety of specialized carrion‐feeding beetles (Kalinová et al., 2009; Peck & Cook, 2017; Watson & Carlton, 2005). Rettenmeyer et al. (2011) suggested that the strong smell of decaying arthropods emanating from the refuse deposits may serve as an attractant chemical cue to many of the visitors, an appealing hypothesis that could be tested by deploying traps containing decaying arthropod corpses at the field site. For instance, we found two species of the genus *Dissochaetus* (Leiodidae) in army ant middens whose members are known carrion feeders that can easily be caught in carrion baited traps (Peck & Cook, 2017). Instead of relying solely on the scent of decaying prey to locate a refuse site, refuse beetles could also use the distinctive smell of the army ants themselves for guidance (Rettenmeyer, 1961). Furthermore, once a colony has been encountered, beetles could follow army ant emigrations to new bivouac sites, where the ants deposit newly acquired prey remains at a fresh disposal site. Indeed, the two most prevalent refuse visitors, aff. *Pridonius* CVB70 and *S. laselva* sp. nov., follow the emigrations occasionally, either on foot or attached to larvae or prey being carried by workers (von Beeren, Blüthgen, et al., 2021). This suggests that these refuse inhabitants already have developed a stronger association with the army ants, possibly even relying on their presence.

Our collection of army ant refuse material yielded a highly variable number of beetles per deposit, with some deposits yielding only a few specimens. Such a low abundance appears to be an artifact of the collection method and unlikely reflects the situation in the wild. Some refuse deposits were partially concealed or difficult to collect, resulting in less refuse material and thus less visitors. Further factors might have influenced the varying visitor abundance such as heavy rain versus dry conditions during collections, or different raiding activities of the army ants (Schneirla, 1971; Teles da Silva, 1982). For instance, it may be counterintuitive that we did not observe a higher abundance of refuse‐visiting beetles in colonies during the statary phase compared to the nomadic phase. While refuse can accumulate over several days in the statary phase, resulting in larger deposits over time (Gotwald Jr, 1995; Kronauer, 2020; Rettenmeyer, 1961; Schneirla, 1971), this phase is characterized by reduced colony activity, with weaker or no foraging raids (Gotwald Jr, 1995; Kronauer, 2020; Schneirla, 1971; Teles da Silva, 1982; Willis, 1967). On the other hand, during the nomadic phase, colonies generally perform longer and larger foraging raids (Gotwald Jr, 1995; Kronauer, 2020; Schneirla, 1971; Teles da Silva, 1982), which supposedly result in a higher volume of fresh prey remains being dumped at the refuse site per day. The similar abundance of refuse‐visiting beetles in both phases of the colony cycle might thus be partly attributed to the trade‐offs between increased persistence but fewer fresh prey remains per day in statary colonies versus ephemeral but elevated prey accumulation in nomadic colonies.

Rove beetles were by far the most common refuse‐visiting beetles, a dominance that was likewise observed in refuse deposits of African *Dorylus* Fabricius, 1793 army ants (Collart, 1934; Paulian, 1948) and *Eciton* Latreille, 1804 army ants in Panama (Rettenmeyer, 1961; Rettenmeyer et al., 2011). This dominance is not surprising, considering that rove beetles are also dominant in soil and leaf litter, and are known to consume animal flesh either as predators or as scavengers (Betz et al., 2018; Thayer, 2005). Furthermore, rove beetles are good dispersers (Betz et al., 2018; Thayer, 2005), and the accumulation of arthropod remains at army ant refuse sites thus must have attracted many of the studied species. In fact, Rettenmeyer observed that more than 100 rove beetles reached a refuse site by flight within half an hour (Rettenmeyer, 1961). Thanks to their small size, their agility, and their defensive chemical weaponry, rove beetles are certainly capable of successfully fending off occasional attacks by ants or other competitors at a refuse site (Parker, 2016; von Beeren, Brückner, et al., 2021). Unfortunately, many refuse‐visiting rove beetles remained unidentified in the present study. We encourage the scientific community to use our voucher material, including specimens, images, DNA barcodes, and DNA extracts, in future taxonomic work.

In addition to the challenges in beetle identification, the life cycles of most *Eciton* associates, including those of refuse visitors and emigration followers, remain entirely unknown (Gotwald Jr, 1995; Rettenmeyer et al., 2011; von Beeren et al., 2016b). We have provided some basic information of beetle life histories by showing that army ant refuse deposits not only serve as a food source for beetles, but also as a breeding ground and a nursery. We frequently observed rove beetles mating, and we detected larvae of 29 beetle species within the deposits. Given the well‐developed legs and relatively large body size of many larvae, at least some might have migrated from the surrounding leaf litter into the deposits, in addition to others hatching from eggs within the deposits. Rettenmeyer (1961) speculated that most of the larvae belong to adults that are also found in the deposits, while some might belong to emigration followers. By rearing larvae to adulthood, Akre and Torgerson (1969) indeed provided a first match between an adult emigration follower and its larvae, an *Eciton*‐associated tachyporine rove beetle of the genus *Vatesus* Sharp, 1876. Many years later, Caterino and Tishechkin (2006) identified a histerid larva in refuse using DNA barcoding. The DNA barcode matched that of an emigration‐following adult, *Paratropinus scalptus* Reichensperger, 1935 (Caterino & Tishechkin, 2006). Adults of this species participate as hitchhikers in the colony emigrations of *E. burchellii* (von Beeren & Tishechkin, 2017). We found larvae of nine additional species in the deposits that are emigration followers as adults, indicating that army ant middens indeed act as a nursery for some of the more obligate guest species. Notably, we did not find a single beetle pupa in our collection. Without the army ants replenishing the middens, especially during the nomadic phase of army ant colonies, the food at a particular refuse site will be depleted within a couple of days (Rettenmeyer, 1961). As a result, beetles that rely on these refuse sites for food, along with most larvae, are likely to disperse in search of an alternative food source or a suitable place for pupation as last instar larvae.

Lastly, as observed by previous authors (Akre & Rettenmeyer, 1966; Gotwald Jr, 1995; Rettenmeyer, 1961), we found a pronounced spatial niche differentiation between species visiting the deposits and those following the colony emigrations. Species common in refuse deposits were rare or absent in army ant emigrations, while most species common in emigrations were rare or absent in refuse sites. This supports the idea that army ant‐associated beetles tend to partition available microhabitats (Akre & Rettenmeyer, 1966; Gotwald Jr, 1995; Rettenmeyer, 1961; von Beeren, Blüthgen, et al., 2021), with some species following the colony emigrations and often living inside the army ants' bivouacs, and others mostly scavenging in the bivouacs' surroundings, especially in the resource‐rich middens.

While quantitative data are mostly lacking, spatial niche differentiation has also been documented among arthropods seeking close contact with other social insects (Hölldobler & Wilson, 1990; Kistner, 1979; Schmid‐Hempel, 1998). For instance, it has been described in the associates of honeybees (Bailey, 1981; Chantawannakul et al., 2016; Morse & Nowogrodzki, 1990), termites (Kistner, 1969, 1979), and other ants (Hölldobler & Kwapisch, 2022; Hölldobler & Wilson, 1990; von Beeren et al., 2011). One example are leaf‐cutter ants, where a diverse fauna of arthropods segregates into distinct microhabitats: some visit refuse piles, some reside as inquilines in fungus chambers, while others interact with foragers outside the nest, such as the infamous ant‐decapitating flies (Eidmann, 1936; Feener & Brown, 1997; Folgarait, 2013). Notably, termites and ants generally host a much higher diversity of guests than bees and wasps (Kistner, 1979; Wilson, 1971). Wilson (1971) attributed this to two key factors. First, bees and wasps generally construct compact, tightly sealed nests in trees. Wilson speculated that this likely limits the number of arthropods preadapted for nest penetration compared to the more accessible, open nests of many ants and termites on or in the ground, where more arthropods reside. Second, refuse, which potential scavengers can feed upon, is scarce around bee and wasp nests. In numerous species, workers merely eject refuse at the nest entrance, where it falls to the ground (Wilson, 1971). In contrast, in leaf‐cutter or army ant societies, refuse is concentrated into large piles, which, as demonstrated here, attract a diverse fauna of visitors.

## 5 CONCLUSION

Army ant colonies attract diverse species, from obligate emigration‐following symbionts to more facultative refuse‐visiting scavengers. Here we showed that army ant refuse deposits, with their nutritionally rich prey remains, attract a diverse assemblage of adult and immature beetles, and thus likely facilitate the completion of these beetles' life cycles. With the present biodiversity inventory, we provide further evidence that army ants foster local diversity in neotropical rainforests and therefore merit special attention in conservation management, ideally by protecting their invaluable habitat (Kronauer, 2020; Pérez‐Espona, 2021).

## AUTHOR CONTRIBUTIONS

**Christoph von Beeren:** Conceptualization (lead); data curation (lead); formal analysis (lead); funding acquisition (lead); investigation (lead); methodology (lead); project administration (lead); software (lead); supervision (lead); validation (lead); visualization (lead); writing – original draft (lead); writing – review and editing (lead). **Sebastian Pohl:** Investigation (supporting); writing – review and editing (supporting). **Martin Fikáček:** Formal analysis (equal); investigation (equal); writing – review and editing (equal). **Stephan Kleinfelder:** Formal analysis (supporting). **Alexey K. Tishechkin:** Formal analysis (equal); investigation (equal); writing – review and editing (supporting). **Shuhei Yamamoto:** Formal analysis (equal); investigation (equal); writing – review and editing (supporting). **Mariana Chani‐Posse:** Formal analysis (equal); investigation (equal); writing – review and editing (supporting). **Dagmara Żyła:** Formal analysis (equal); investigation (equal); writing – review and editing (supporting). **Alexandra Tokareva:** Formal analysis (supporting); investigation (equal); writing – review and editing (supporting). **Munetoshi Maruyama:** Formal analysis (equal); investigation (equal); writing – review and editing (supporting). **W. Eugene Hall:** Formal analysis (equal); investigation (equal). **Liliana P. Sandoval:** Formal analysis (equal); investigation (equal); writing – review and editing (supporting). **Daniel J. C. Kronauer:** Conceptualization (lead); funding acquisition (supporting); investigation (equal); methodology (equal); project administration (supporting); resources (supporting); supervision (supporting); writing – review and editing (lead).

## FUNDING INFORMATION

C.v.B. was supported by grants from the German Science Foundation (DFG; BE5177/1‐1, BE5177/4‐1, and BE5177/4‐2), the National Geographic Society's Committee for Research and Exploration, and a Bristol‐Myers Squibb Postdoctoral Fellowship from The Rockefeller University. The work of M.F. was supported by the Ministry of Culture of the Czech Republic (DKRVO 2019–2023/5.I.e, National Museum, 00023272). S.Y. was supported by a Grant‐in‐Aid for JSPS Fellows (no. 20J00159) given by the Japan Society for the Promotion of Science (JSPS), Tokyo, Japan. D.Ż. and A.T. were supported by the Polish National Science Centre (grant no. 2019/35/B/NZ8/03431). D.J.C.K. was supported by a Carl & Marian Rettenmeyer Ant‐Guest Endowment Award and is an Investigator of the Howard Hughes Medical Institute.

## CONFLICT OF INTEREST STATEMENT

The authors have no competing interests.

## BENEFIT‐SHARING STATEMENT

Benefits from this research accrue from the sharing of our data and results on public databases. In previous research (von Beeren et al., 2016b), we have deposited beetle specimens at the Escuela de Biología of the University of Costa Rica. We will additionally deposit a representative collection of the specimens studied here at this institution after final processing (further identification, cleaning, mounting and labeling). Updates on depository changes can be traced via the BOLD Systems database.

## Supporting information


Appendix S1.

Appendix S2.

Appendix S3.

## Data Availability

Sequences are deposited in GenBank and the BOLD Systems (for accession numbers see Appendix S1—Specimen information). Voucher specimens are deposited at eight registered museum collections and at the TU Darmstadt Insect Collection (Appendix S1—Specimen information). DNA extracts are deposited at the TU Darmstadt Insect Collection. Specimen images are deposited at BOLD Systems (Appendix S1—Specimen information).
