# Supplementary material for: Army ant middens – Home and nursery of a diverse beetle fauna
Source: Ecol Evol. 2023 Sep 19;13(9):e10451. doi: 10.1002/ece3.10451 (PMC10509147; doi:10.1002/ece3.10451)
Supplement: Supplementary file 2 — Appendix S2. [file ECE3-13-e10451-s003.pdf]

Ecology and Evolution
Open Access

Appendix 2

Army ant middens – home and nursery of a diverse beetle fauna

Authors: Christoph von Beeren*, Sebastian Pohl, Martin Fikáček, Stephan Kleinfelder, Alexey K. Tishechkin, Shûhei Yamamoto, Mariana Chani-Posse, Dagmara Żyła, Alexandra Tokareva, Munetoshi Maruyama, W. Eugene Hall, Liliana P. Sandoval, Daniel J.C. Kronauer*

*Correspondence to:
Christoph von Beeren: cvonbeeren@gmail.com
Daniel J.C. Kronauer: dkronauer@rockefeller.edu

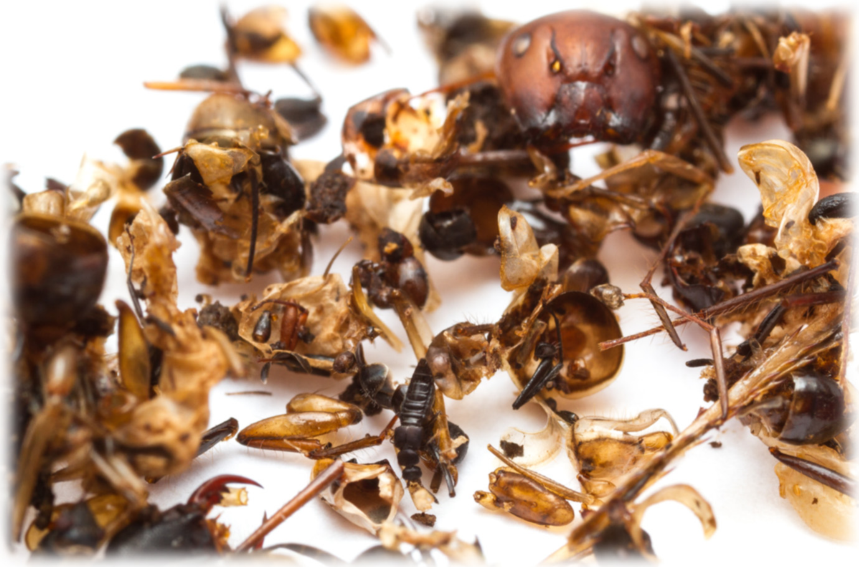

Image credit: D. Kronauer

Appendix 2 – Table of Content:

- **Species identification/description**
- **PCR primer combinations used in this study.**
- **Supplemental reference list**

Species identification/description

In this section we provide information about the morphological identification of some of the studied species. Morphological identifications were exclusively carried out with adult beetles, while identifications of beetle larvae were solely based on *COI* barcode data. Morphological identification of many specimens was problematic, partly because the taxonomy of some groups is not well resolved, and partly due to lack of time resources for identification. As a result, we often provided merely subfamily, tribal, or genus names followed by a unique identifier (e.g., *Coproporus* CVB11). We are aware that such imprecise species denominations impede the affiliation to biological information that might be obtained in future work. To bridge this gap, we deposited voucher material consisting of DNA barcodes, DNA extractions, specimen images, and the actual specimens. The DNA barcodes might be particularly helpful for future comparisons because this character can be easily matched across studies, and specimen deposition allows for verification of defined species boundaries using morphological characters. Several of the refuse-visiting beetles most likely represent yet undescribed species (e.g., aff. *Pridonius* CVB09, aff. *Pridonius* CVB70, Xantholinini CVB10, Phelister CVB85, Medonina CVB52, and Medonina CVB53). However, a comprehensive analysis of all refuse-visiting beetles was beyond the scope of the present work. We encourage the scientific community to integrate the deposited voucher specimens in future taxonomic work. Focus-stacked images of all the species described below are deposited at BOLD systems and can be found by using the sample ID as search term (Appendix 1 – Specimen information).

Staphylinidae

The rove beetles (Staphylinidae) were the most abundant and most diverse group of refuse-visiting beetles. Many staphylinid species remained unidentified, particularly in the megadiverse subfamily Aleocharinae. The taxonomic revisions of Seevers (Seevers, 1965) and Kistner & colleagues (Jacobson & Kistner, 1998; Kistner & Jacobson, 1990; Kistner & Mooney, 2011) allowed us to identify some of the more typical army ant-associated beetles, and their genus and species keys provided a baseline for identifications.

Staphylinidae – Osoriinae

***Mimogonus fumator* (Fauvel, 1889) & *Geotrochopsis pubescens* Irmler 2016:** Both species were identified by Ulrich Irmler. *Mimogonus fumator* was identified based on external morphology (Irmler, 1981), while the aedeagus was additionally dissected in *Geotrochopsis pubescens* (see Irmler, 2016). The head of the *G. pubescens* specimen was lost during transport but it is still visible on the voucher image (see BOLD Systems).

Staphylinidae – Tachyporinae

***Vatesus* cf. *clypeatus* CVB72:** *Vatesus* beetles of *Eciton* army ants at LSBS were previously analyzed (von Beeren et al., 2016b; von Beeren, Blüthgen, et al., 2021), and we were therefore able to reliably identify the species collected in refuse deposits using DNA barcodes as well as larval coloration patterns (von Beeren et al., 2016b).

***Coproporus*:** We frequently found limuloid, blackish shining, tachyporine beetles in *Eciton* refuse deposits. We consider them to belong to the genus *Coproporus*, although they have some unusual characters (e.g., presence of the secondary lobes of the tergite IX in males, as seen in *Cilea*; Yamamoto, 2021). DNA barcodes clustered the specimens into four distinguishable genetic clusters (BIN:AEQ9500; BIN:AET7799; BIN:AEU6410; BIN:AEU6411). We dissected aedeagi and the abdominal terminalia of specimens belonging to these four clusters but were not able to morphologically distinguish all of the genetic clusters. We were not able to distinguish *Coproporus* CVB12 (BIN:AEU6411; dissected specimens: 3 males, 1 female) from *Coproporus* CVB81 (BIN:AET7799; dissected specimens: 2 males) and the same applied to the species couple *Coproporus* CVB11 (BOLD:AEQ9500; dissected specimens: 4 males, 5 females) and *Coproporus* CVB71 (BOLD:AEU6410; dissected specimens: 2 males, 3 females). Although the external morphology of all four clusters was quite similar, we were able to distinguish the species pair *Coproporus* CVB11/CVB71 from species pair CVB12/CVB81. These two species pairs were distinguishable, because *Coproporus* CVB12/CVB81 had narrower and longer outer lobes with shallower medial large emargination in the male sternite VIII in comparison to that of CVB11/CVB71 (Fig. S1). Besides, the second inner lobes in the male sternite VIII of CVB12/CVB81 were narrower and longer than those of CVB11/CVB71 (Fig. S1). Furthermore, the medial notch of the inner (median) lobes in the male tergite VIII was much deeper in CVB11/CVB71 than in CVB12/CVB81 (Fig. S1). Additionally, the structures of the internal sclerites in the median lobe of the male aedeagus were clearly distinct (see Fig. S1).

Figure S1. Sternites VIII and tergites VIII of females and males, as well as aedeagi of males, of four distinct *Coproporus* species. Images were taken with a Zeiss Axiolab 5 equipped with an Axiocam 208 color. Sample IDs are given for each image (see also Appendix 1- Specimen information). A high-resolution version of the figure has been deposited at Zenodo (doi:10.5281/zenodo.8199007).

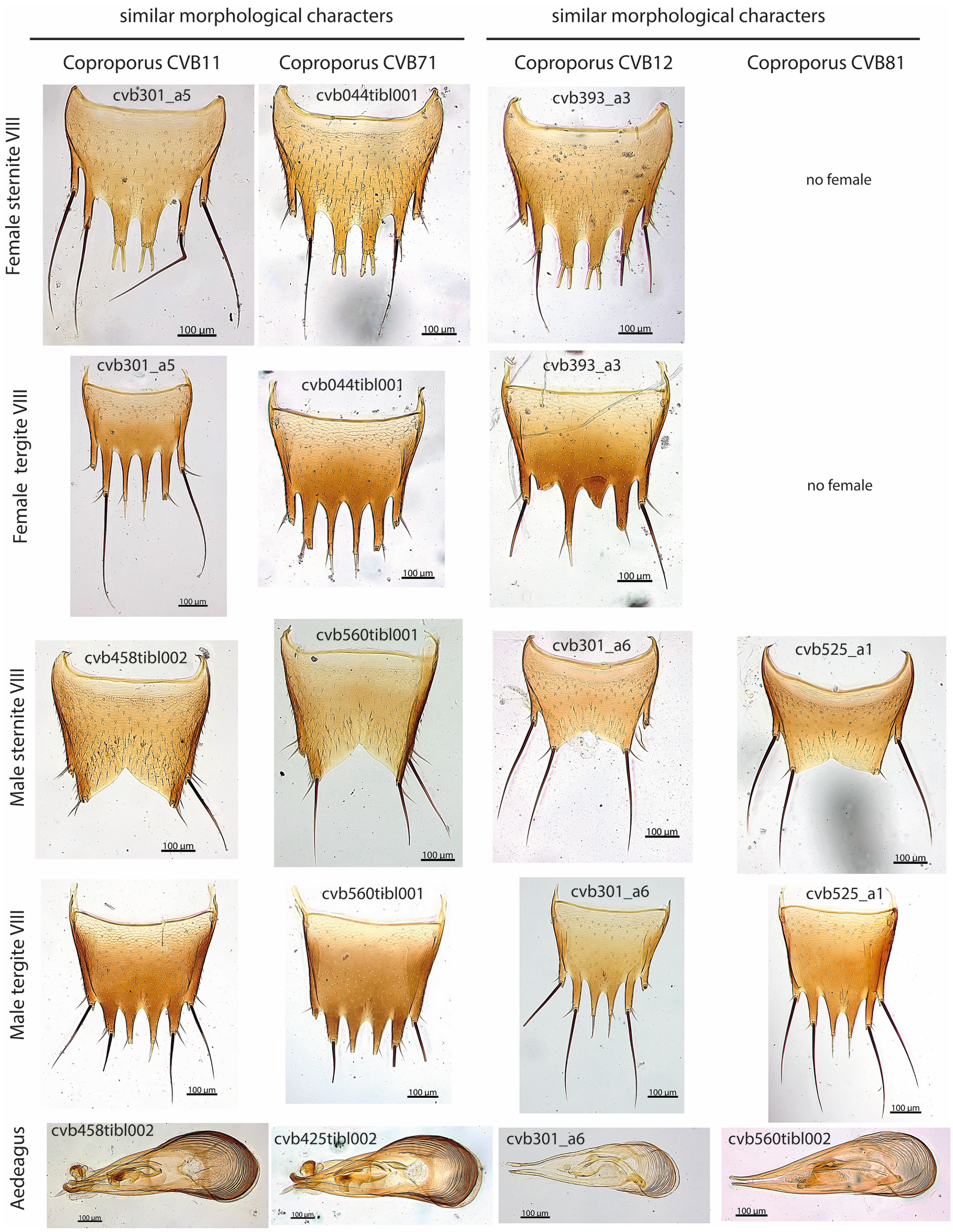

Staphylinidae – Staphylininae

aff. *Pridonius* CVB09 & aff. *Pridonius* CVB70: Both species belong to the so-called 'Neotropical lineage' within the subtribe Philonthina (Staphylininae, Staphylinini), and they are representatives of a new, yet undescribed genus that is near to *Pridonius iheringi* (Chani-Posse et al., 2018). All inspected adults (aff. *Pridonius* CVB09: N = 1 specimen; aff. *Pridonius* CVB70: N = 6 specimens) agree with the combination of characters for this new, yet undescribed genus (Chani-Posse et al., 2018). In the following we give a brief preliminary diagnosis of relevant generic characters: the head is slightly to moderately wider anteriad, eyes are larger than tempora, antennae with the third segment is longer than second segment (a3/a2 ratio > 1.5), disc of prothorax is coarsely punctated, sternopleural suture is distinctly oblique, mesocoxae are at least narrowly separated, metatarsus with the first segment is not shorter than fifth segment and the abdomen is of subconical shape. Members of the new genus resemble *Pridonius iheringi* (Bernhauer) because of the following characters: head with gular sutures joined posteriorly before neck, protarsus with the first four segments more or less flattened dorso-ventrally and metacoxae without ventral spines. However, they clearly differ from *Pridonius iheringi* by having other characters which are common within Philonthina, leaving no doubt on their affiliation to this subtribe. One such character is the superior marginal line of hypomeron distinctly deflexed under the anterior angles of prothorax, which appears not deflexed in *Pridonius iheringi*. This condition (among others) led previous researchers to consider the latter species within the subtribe Quediina (Chani-Posse et al., 2018). The abovementioned taxa are part of an on-going revisionary study (Rodriguez-Melgarejo & Chani-Posse, in prep.).

Xantholinini CVB10: This species belongs to Xantholinini (Staphylininae), and it likely represents a new species and may, in addition, represent a new, yet undescribed genus, with intermediate characters between *Gyrohypnus* Leach and *Lissohypnus* Casey (Navarrete-Heredia et al., 2002; Smetana, 1982). In the following we give a brief preliminary diagnosis of relevant generic characters: eyes moderately shorter than tempora seen from above (e/t ratio > 0.8), ocular puncture bearing long seta far from inner margin of eye, distance separating ocular punctures from each other not more than 2.5 times distance separating each ocular puncture from inner margin of eye, last segment of maxillary palpus fusiform, at widest point almost as wide as the preceding segment, neck at least one fourth as wide as head, pronotal disc with two dorsal rows of punctures, superior line of pronotal hypomeron not deflexed until close to anterior angles and not joining inferior line, metatibia with both subapical and apical ctenidia. Characters common to this putative new taxon, *Gyrohypnus* and *Lissohypnus* are the last segment of maxillary palpus fusiform as well as the presence of two dorsal rows of punctures on the pronotum and the superior line of pronotal hypomeron not deflexed until close to anterior angles and not joining inferior line. Among other characters, it resembles *Lissohypnus* but differs from *Gyrohypnus* by the position of the ocular puncture (close to inner margin of eye in *Gyrohypnus*). Likewise, it resembles *Gyrohypnus* but differs from *Lissohypnus* by the metatibia having both subapical and apical ctenidia as opposed to that of *Lissohypnus* with only an apical ctenidium.

Staphylinidae – Paederinae

All specimens of Paederinae belong to the tribe Lathrobiini and the so-called 'Medonina and allied taxa' lineage (sensu Żyla et al., 2019). They would all be classified to the subtribe Medonina, but as recent studies showed, the subtribe is polyphyletic and is in urgent need of revisionary work (Żyla et al., 2021). Nevertheless, some common characters are shared between different lineages of Medonina, which helped to assign them to this artificial subtribe. The characters are as follows: setae between ommatidia, neck wider than 1/3 of head width, protibial combs placed longitudinally, hind tibia with ctenidium on one side only. Further identification was done with the help of the Paederinae key of Navarrete Heredia *et al.* (2002).

Achenomorphus **CVB04:** This species was identified as a member of the genus *Achenomorphus* Motschulsky by the following characters: head and pronotum densely punctured with very small umbilicate punctures, pronotum with mid-longitudinal impunctate line; pronotum trapezoidal, distinctly wider anteriorly.

Lithocharis **species:** We identified several species as members of the genus *Lithocharis* Dejean, 1833. However, all *Lithocharis* specimens lack some characters typical for the Holarctic species of the genus, and the assignment should be treated as temporary. Neotropical *Lithocharis* species require revisionary taxonomic work. The species *Lithocharis* CVB05, *Lithocharis* CVB07, and *Lithocharis* CVB08 were classified as members of this genus due to the following characters: head and pronotum very finely and densely punctate or sculptured, without obvious umbilicate punctures, gular sutures separate, disc of head, pronotum and elytra generally moderately to densely punctate and setose. We identified one species as *Lithocharis* cf. *discoidales*, which closely resembles the species *Lithocharis discoidalis* Sharp, 1886 based on the habitus. It was also compared with the type specimen (kept in the Natural History Museum in London, NHMUK). Since the type specimen is a female and thus lacks diagnostic characters important to distinguish species in this group, it was not possible, however, to unambiguously confirm the species identification.

Medonina CVB52 & Medonina CVB53: These two remaining species could not be assigned to any known genus and possibly represent an undescribed genus. The most characteristic feature is a smooth strip posteriorly in the middle of head and head emargination at the base. Similar characters are observed in *Ecitomedon* Bernhauer, 1925, but based on the other characters, the two species could not be classified to this genus. In the key of Navarrete Heredia *et al.* (2002), the two species key out as "*Medon* generic complex".

Staphylinidae – Aleocharinae

Tribe Ecitocharini: Four species of refuse-visiting beetles belonged to the tribe Ecitocharini: *Ecitochara connexa* Reichensperger 1933, *Ecitophya simulans* Wasmann 1889, *Ecitomorpha* cf. *nevermanni* Reichensperger 1935, and *Ecitomorpha* cf. *breviceps* Reichensperger 1933. Except for *Ecitochara connexa*, the species were analyzed previously by the authors and DNA barcodes as well as morphological characters distinguishing the species were described (von Beeren et al., 2018). *Ecitochara connexa* was identified using the species key of Kistner & Jacobson (1990). It seems to be a rare species as previous collection efforts failed to detect this species (von Beeren et al., 2018; von Beeren, Blüthgen, et al., 2021). However, one specimen was previously detected at La Selva by Munetoshi Maruyama in 2015 (unpublished data).

Tetradonia: *Eciton*-associated *Tetradonia* species at LSBS were previously processed by us showing that species can be distinguished based on morphological characters and DNA barcodes (von Beeren et al., 2016a). Noteworthy, among the species occupying several microhabitats created by army ant colonies was *Tetradonia* cf. *marginalis*, an aleocharine rove beetle that was found in colony emigrations, army ant raids, and army ant refuse deposits (Akre & Rettenmeyer, 1966; von Beeren et al., 2016a, von Beeren, Blüthgen et al., 2021). This predatory species inhabits the area around the bivouac, and attacks and overwhelms army ant workers during raids and emigrations (Akre & Rettenmeyer, 1966; von Beeren et al., 2016a, von Beeren, Blüthgen et al. 2021). Akre & Rettenmeyer (1966) noted that *Tetradonia marginalis* is among the most common rove beetle in *E. burchellii* refuse deposits. The present work disagrees with these results because we only detected two *T.* cf. *marginalis* specimens at *E. burchellii* refuse dumps, while they were abundant in emigrations and raids (von Beeren, Blüthgen et al. 2021). Noteworthy, Akre & Rettenmeyer`s study (1966) took place in a different locality (mostly Panama), and it is uncertain whether the studied beetles constitute the same species as those studied in Panama (see von Beeren et al., 2016a). At LSBS at least, our results favor a pronounced spatial niche separation in this species.

Histeridae

Many army ant-associated histerid beetles of the subfamily Haeteriinae are relatively easy to identify based on their external morphology. Two published keys for members of the subfamily Haeteriinae can be used to identify the genera (Helava, et al., 1985; Tishechkin, 2007). All but eight species were previously identified, and the relevant literature cited (von Beeren, Blüthgen, et al., 2021). The identification of the following histerid taxa is based on comparison of our specimens with authentic material, i.e., type series or/and specimens identified by a species describer: *Paratropinus scalptus* Reichensperger, 1935, *Neocolonides howdeni* Dégallier 1998, *Synoditulus debilis* Reichensperger, 1938, *Trichoreninus geminus* (Reichensperger, 1935). These comparisons revealed complete matches in external and male genital morphology. Two species were only identified to their genus (*Paratropinus* CVB96; *Phelister* CVB85). *Phelister* CVB85 certainly represents an undescribed species from the informal species group '*P. sanguinipennis group*' (see Caterino & Tishechkin, 2020). The herein collected male specimen will be considered in an upcoming revisionary work of the group. We morphologically identified the second species (*Paratropinus* CVB96) as a member of the genus *Paratropinus* based on morphology, although

DNA barcode similarity between the specimen and the *Paratropinus scalptus* specimens was only 94%. Despite a relatively recent generic-level revision of the tribe Nymphisterini, the tribe where this species belongs (Tishechkin, 2007), the accumulation of recently collected material regularly brings undescribed taxa, both at the species and at the generic levels. Unfortunately, this single specimen is a female, and male genital characters are needed in this tribe for a reliable assignment to genera. Therefore, the generic assignment remains tentative. *Plagiogramma* histerids (species in this work: *Plagiogramma* CVB61, *Plagiogramma schmidti* (Wenzel & Dybas, 1941)) are members of the subfamily Tribalinae, and they are thus not included in the mentioned generic keys of Haeteriinae. There is no published species key at the moment to solidly identify the specimens from this genus for the neotropical Region. *Plagiogramma* CVB61 clearly belongs to *Plagiogramma*, and it is easily distinguishable from the other two neotropical tribaline genera by the presence of additional transverse stria on mesosternum. The genus is a subject of ongoing revisionary study by P. W. Kovarik and our species identifications thus remain tentative.

Rhysodidae

***Clinidium erwini* Bell & Bell 2009:** We used the species key and characters provided by Bell & Bell (2009) to identify the species. The shape of the linear median grove of the pronotum and the shape of the head lobes allowed the identification as *Clinidium erwini*, also separating it from the close species *C. rossi*. Note that this beetle was probably not attracted to the army ants' prey remains but was more likely by chance present at or close to the refuse site. *Clinidium erwini* Bell & Bell 2009 is a member of the carabid subfamily Rhysodinae (Vasilikopoulos et al., 2021), which live in dead wood, where they consume slime molds using their highly modified mouthparts (Bell & Bell, 2009).

Carabidae

Trechinae CVB28: This carabid beetle was identified by Fabian Bötzl as a member of the subfamily Trechinae, because it has the characteristic intrusion at the end of the elytra (in German 'Trechusbogen').

Scarabaeidae

***Onthophagus limonensis* Kohlmann & Solís, 2001:** We used the species key of *Onthophagus* species of Costa Rica by Kohlmann & Solís, (2001) to identify this species. A 100% DNA barcode match to a specimen uploaded by Angel Solis gave us additional confidence that the species ID is correct.

Leiodidae

We found two species of leiodid beetles. Both species were members of the genus *Dissochaetus* because of their serrate margins of the inner apical metatibial spurs, which are usually more than a half of the length of the first metatarsomere (Gnaspini, 1993). *Dissochaetus* beetles occur in the Neotropics and are known as scavengers at carrion and dung, sometimes troglophiles in caves, and a few specimens have been previously collected in *Eciton* refuse deposits (Peck & Cook, 2017; Peck et al., 2020).

Dissochaetus hetschkoi **Reitter, 1885**: This species was identified as *D. hetschkoi* by the following characters of the male genitalia: median lobe of the aedeagus elongate and broad, apex narrow and long, with a pair of lateral setae. Internal sac inverted, with paired, irregularly shaped sclerites, patches of small spines apically. Parameres elongate, narrow, inwardly curved apically and the apex with a small toothlike process. Genital segment with the apex of the tergite shallowly emarginated, with a few setae; lateral lobes rounded apically with apical setae; ventral lobes of pleurites narrow, inwardly curved, with apical setae; sternite present, short, and broad bearing many setae apically. This species was previously reported from our study site, i.e., La Selva Biological Station in Costa Rica (Peck & Cook, 2017).

Dissochaetus confusus **Salgado-Costas, 2010**: We identified the single specimen of this species by the following characters: median lobe of the aedeagus triangular with long, narrow apex and a pair of lateral setae. Internal sac with patches of small spines; medially and basally with sclerotized structures. Parameres extending far beyond apex of the median lobe, curved basally and in apical third, small dentiform structure medially at apex; two elongate apical setae. Genital segment with the apex of the tergite rounded and with apical setae; lateral lobes of pleurites with long apical setae; ventral lobes of pleurites bearing long apical seta and row of inwardly directed setae on inner margins; sternite short, bearing apical setae. Again, this species was previously reported from our study site, i.e., La Selva Biological Station in Costa Rica (Peck & Cook, 2017).

Hydrophilidae

In the following we provide formal species descriptions of the refuse-visiting beetles *Cercyon pohli* sp. nov and *Sacosternum laselva* nov. sp.. Museum abbreviations are as follows: BMNH = Natural History Museum, London; NMPC = National Museum of Prague.

Cercyon pohli Fikáček sp. nov.

Habitus images and relevant characters are illustrated in Figure S2 (a-g) of this Appendix.

Type material. Holotype: male (NMPC): 'COSTA RICA: Heredia Prov., La Selva Biol. Station, 24.ii.2013, 10°25.838205'N 84°0.418470W, refuse deposit of *Eciton burchellii*, leg. C. v. Beeren & S. Pohl, Colony ID: EB01, sample ID: cvb117roun001, *COI* GenBank accession number: OQ173065'. **Paratypes** (NMPC, BMNH): 1 male: 'COSTA RICA: Heredia Prov., La Selva Biol. Station, 19.ii.2013, GPS: 10°25.838205'N 84°0.418470'W, refuse deposit of *Eciton burchellii,* leg. C. v. Beeren & S. Pohl, Colony ID: EB01, sample ID: cvb103roun001'; 1 male: 'COSTA RICA: Heredia Prov., La Selva Biol. Station, 14.ii.2013, GPS: 10°25.838205'N 84°0.418470W, refuse deposit of *Eciton burchellii*, leg. C. v. Beeren & S. Pohl, Colony ID: EB01, sample ID: cvb055roun001'; 1 spec.: 'COSTA RICA: Heredia Prov., La Selva Biol. Station, 24.ii.2013, 10°25.838205'N 84°0.418470'W, refuse deposit of *Eciton burchellii*, leg. C. v. Beeren & S. Pohl, Colony ID: EB01, sample ID: cvb117roun003; 1 spec.: 'COSTA RICA: Heredia Prov., La Selva Biol. Station, 17.iii.2013, GPS: 10°25.838205'N 84°0.418470'W, refuse deposit of *Eciton burchellii*, leg. C. v. Beeren & S. Pohl, Colony ID: EB03, sample ID: cvb377roun001'. *COI* GenBank accession numbers of paratypes are given in Appendix S1 – Specimen information.

Description. Body rounded, globular, widest at midlength, moderately convex in lateral view (Figs 4a–c). Body length 1.9–2.4 mm (holotype: 2.3 mm), body width 1.2–1.3 mm (holotype: 1.3 mm); TL/TW ratio = 1.7. ***Coloration.*** Dorsal side of pronotum and head reddish brown, clypeus slightly paler than rest of head, elytra darker, brown to dark brown; ventral side brown; coxae, femora, tibiae, mouthparts, antennae, and tarsi reddish brown. ***Head.*** Clypeus with moderately dense punctation consisting of small semicircular punctures; interstices without microsculpture; anterior margin of clypeus slightly convex. Frons with moderately dense punctation consisting of small semicircular punctures; interstices without microsculpture. Eyes moderately large, separated by 4.5× of width of one eye. Mentum 1.9× wider than long, subrectangular in shape; anteromedian part impressed; surface with very sparse punctation consisting of small punctures; interstices without microsculpture. Maxillary palpomere 2 slightly longer than palpomere 3 and 4 each. Scapus ca. as long as antennal club; club slightly widening from base towards apex. ***Prothorax.*** Pronotum as convex as elytra in lateral view, evenly arcuate, without any impressions. Posterolateral corners rectangular; lateral margin arcuate, with narrow lateral rim. Pronotal punctation as dense, as large and of the same shape as on frons, interstices with much smaller punctures between main punctures here and there, punctures without setae; interstices without microsculpture. Transverse row of punctures on posterior margin of pronotum absent. Prosternum carinate medially, carina narrow, slightly projecting anteriad, straight in lateral view; posteromesal projection pointed, without median notch; prosternum in front of procoxae rather short. Lateral margins of antennal grooves subangular, antennal grooves reaching ca. half-width of hypomeron.

Mesothorax. Scutellar shield bearing few small punctures, interstices without microsculpture. Elytron with 10 longitudinal series of punctures; series 1–5 arising basally, series 6–10 getting gradually less and less apparent anteriorly, indistinguishable from ground punctation anterolaterally. Serial punctures moderately large, rounded, moderately dense, ca. 2× larger than interval punctures, not connected by longitudinal stria. Elytral intervals flat; series not impressed on elytral disc, getting indistinctly impressed posteriorly and laterally. Interval punctation irregular except for lateral-most intervals, consisting of very small punctures, each bearing short seta. Inner pubescent part of epipleura slightly narrower than outer glabrous one; glabrous part inclined. Preepisternal plate narrow (Fig. S2d), 4× longer than wide, spindle-like, flat, bearing very sparse and fine punctures, interstices without microsculpture; plate narrowly overlapping the anterior margin of metaventrite. ***Metathorax.*** Metaventrite slightly longer than preepisternal elevation of mesothorax (Fig. S2d); its median portion without microsculpture, bearing fine sparse punctation; lateral portions with dense microsculpture, with some intermixed longer setae. Anterolateral ridge arcuately bending posteriad towards lateral margin of metaventrite, running very close to anterolateral corner. Femoral lines absent. Anepisternum 5.0× longer than wide. ***Legs.*** Tibiae of all legs with sparse lateral spines, tarsi with slightly longer setae ventrally. ***Abdomen.*** Ventrites 1 with median longitudinal carina, other ventrites without ridges; posterior margin of ventrite 5 entire. ***Male genitalia*** (Figs 4e–g). Tegmen plus parameres 0.68 mm long; median lobe 0.60 mm long. Phallobase 2.9× longer than parameres, conical, parallel-sided, slightly asymmetrical basally, without basal projection. Parameres parallel-sided in basal 3/5, narrowing between apical 1/5 to 2/5, with slightly expanded membranous part apically; apex with two short setae. Median lobe nearly parallel-sided, arcuately pointed; gonopore moderately large, transverse; basal apodemes long and slender. Median portion of sternite 9 nearly as long as lateral struts, partly membranous, pointed apically, with two closely aggregated setae on apex.

Diagnosis. By the combination of the small body size, dorsal coloration without darker spots, absence of femoral lines, and the form of the aedeagus, *C. pohli* is very similar to *C. integer* Sharp, 1882 (Sharp, 1882). The specimens from Costa Rica examined by us from Guanacaste and Puntarenas provinces, compared to types from Mexico and Guatemala, differ from *C. pohli* sp. nov. in (1) distinctly impressed elytral series, (2) much smaller eyes with eyes separated by 6× the width of one eye, (3) wider aedeagus with relatively shorter parameres (1/4× the length of phallobase), and (4) very narrow median portion of male sternite 9. Other small-sized Central American species without femoral lines can be distinguished from *C. pohli* by larger dark body and aedeagus with long paremeres with widely expanded apex (*C. ebeninus* Sharp, 1882), or by pronotum with central dark spots (*C. variegatus*, see Arriaga-Varela et al., 2017).

Etymology. The new species is dedicated to Sebastian Pohl, then a research assistant of the first author, who helped to collect the *Eciton* refuse material in which the described specimens of this species were found.

Phylogenetic placement. The molecular analysis by Arriaga-Varela *et al.* (2021) revealed that *Cercyon* is a largely polyphyletic genus in need of reclassification. Since this was not yet done, we are assigning the species to *Cercyon* where it would belong based on the morphology of its mesoventral plate and prosternum. The analysis of the available *COI* barcodes did not reveal a reliable placement in the tree by Arriaga-Varela *et al.* (2021): the barcode goes to members of the

clade XI (largely Neotropical) when clustered by similarity but reveals in clade VII (Palaearctic *Cercyon*) when analyzed using ML likelihood; the former placement is quite probable, the latter seems improbable, since no member of clade VII occurs in the Neotropics. The generic placement will need to be corrected once the Neotropical '*Cercyon*' is divided to monophyletic genera, and once additional sequences of this species will be acquired.

Biology. Being present in nine refuse deposits of *Eciton burchellii*, *Cercyon pohli* was among the more common species visiting these microhabitats. No other information about its biology is known yet.

Distribution. So far only known from the type locality, the La Selva Biological Station in Costa Rica.

Figure S2. Morphological characters of new water scavenger beetle species (Hydrophilidae). (a-g) *Cercyon pohli* sp. nov.: Shown are (a-c) habitus images (a, dorsal; b, lateral; c, ventral), (d) detail of mesoventral plate and metaventrite (only right side), (e-g) male genitalia (e, sternite 9; f, tegmen; g, median lobe). Scale bars apply to a–c (1 mm) and e–g (0.25 mm). (h-o) *Sacosternum laselva* sp. nov.: Shown are (h-j) habitus images (h, dorsal; i, ventral; j, lateral), (k) detail of the prosternal plate (arrow: lateral expansion of the plate), (l) mesoventral plate and metaventrite (right side only; arrow: lateral triangular area on the mesoventrite), (m) abdominal ventrites, and (n-o) male genitalia (n, general view; o, detail of the apex of the median lobe). Scale bars apply to h-j (1 mm) and n (0.25 mm). A high-resolution version of the figure has been deposited at Zenodo (doi:10.5281/zenodo.8199007).

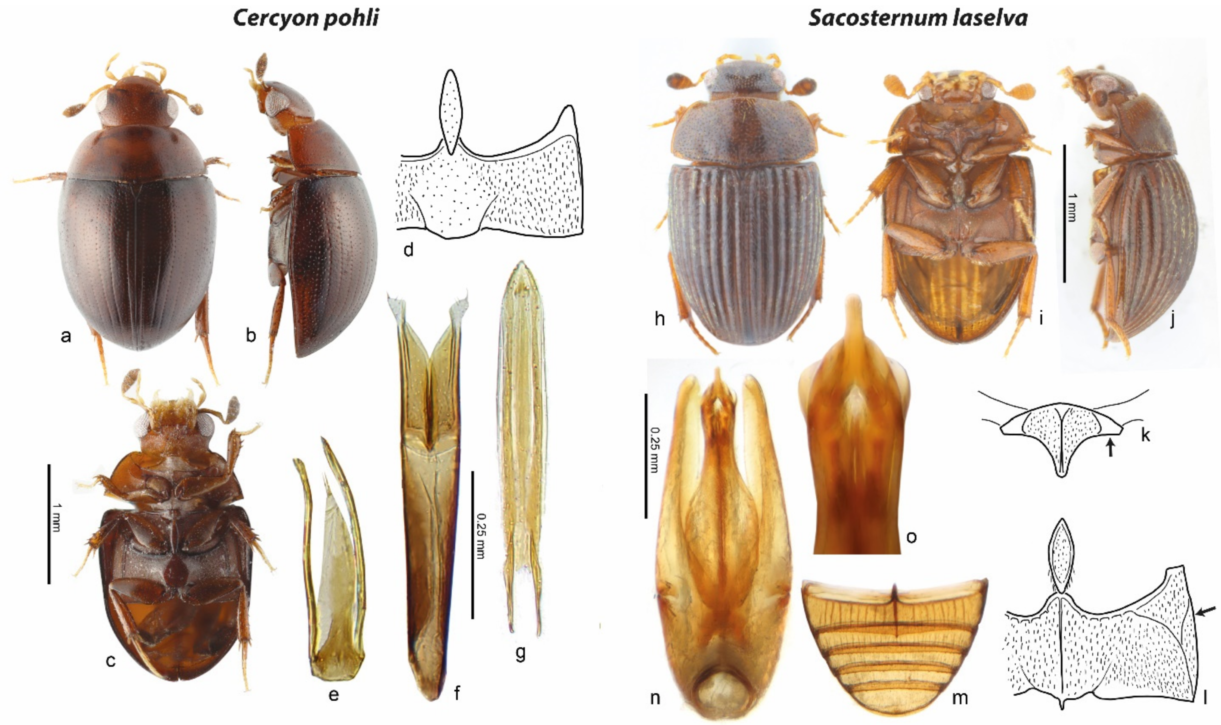

Sacosternum laselva Fikáček sp. nov.

This species was previously analyzed in a molecular phylogenetic study (as *Sacosternum* sp.; Arriaga-Varela et al., 2021) and in two studies addressing the host specificity and integration mechanisms of *Eciton* guests (as *Sacosternum* aff. *lebbinorum*; von Beeren, Blüthgen, et al., 2021; von Beeren, Brückner, et al., 2021). Habitus images and relevant characters are illustrated in Figure S2 (h-o).

Type material. Holotype: 1 male (NMPC): 'COSTA RICA: Heredia Prov., La Selva Biol. Station, 9.ii.2013, 10°25.847'N 84°0.404'W, collected in emigration of *Eciton burchellii foreli*, leg. C. v. Beeren, colony ID: EB01, sample ID: cvb015miro001, *COI* GenBank accession number: MW129390'. **Paratypes** (NMPC, BMNH): 1 male (NMPC): 'COSTA RICA: Heredia Prov., La Selva Biol. Station, 7.iv.2013, 10°25.847'N 84°0.404'W, in refuse deposit of *Eciton burchellii foreli*, leg. C. v. Beeren & S. Pohl, colony ID: EB09, sample ID: cvb548miro001-2'; 1 female (NMPC): 'COSTA RICA: Heredia Prov., La Selva Biol. Station, 8.iv.2013, 10°25.847'N 84°0.404'W, in emigration of *Eciton burchellii foreli*, leg. C. v. Beeren & S. Pohl, colony EB11, Cvb572miro001'. *COI* GenBank accession numbers of paratypes are given in Appendix S1 – Specimen information and in (von Beeren, Blüthgen, et al., 2021).

Description. Body elongate oval, widest ca. at midlength, weakly convex in lateral view (Fig. S2h – j). Body length 1.9–2.0 mm (holotype: 2.0 mm), body width 1.1–1.2 mm (holotype: 1.2 mm); TL/TW ratio = 1.8. ***Coloration.*** Dorsal side dark brown; ventral side dark reddish brown; coxae, femora, tibiae, mouthparts, antennae, and tarsi reddish brown. ***Head.*** Clypeus with moderately dense punctation consisting of large semicircular punctures, each puncture bearing fine decumbent seta; interstices without microsculpture; anterior margin of clypeus slightly concave. Interocular area with triangular area defined by shallow depressions. Frons with moderately dense punctation consisting of large semicircular punctures; interstices without microsculpture. Eyes moderately large, separated by 3.5× of width of one eye. A tuft of yellowish erect setae anteriorly of each eye absent. Mentum 2.6× wider than long, pentagonal in shape; anteromedian part slightly impressed; surface with sparse punctation consisting of small punctures; interstices without microsculpture. Maxillary palpomeres 2 and 4 ca. twice as long as palpomere 3. Scapus slightly shorter than antennomeres 2–6 combined. ***Prothorax.*** Pronotum slightly more convex than elytra in lateral view, weakly triangular on posterior margin; surface shallowly sulcate, bearing very shallow oblique impression posteriorly on each side. Posterolateral corners forming obtuse angle; lateral margin angulate, with wide and distinct lateral rim. Pronotal punctation as dense as that on frons, consisting of moderately large semicircular punctures; interstices without microsculpture. Transverse row of punctures on posterior margin of pronotum clearly defined, consisting of slightly larger circular punctures. Prosternal plate 1.75× wider than long, lateral extensions present (median part 1.2× wider than long; Fig. S2k); posteromesal projection with shallow notch; median carina of prosternum projecting anteriad mesally, straight in lateral view. Lateral margin of antennal grooves circular. ***Mesothorax.*** Scutellar shield bearing few moderately large punctures, interstices without microsculpture. Elytral series 1–7 arising basally, series 8 and 9 arising subbasally, joining each other anteriorly. Serial punctures small, transverse, sparsely arranged, as larger as interval punctures. Elytral intervals moderately convex at suture, becoming strongly convex (nearly ridge-like) laterally and posteriorly; series deeply impressed mesally and laterally. Interval punctation arranged in series at least on some intervals, consisting of small, transverse scar-like punctures.

Inner pubescent part of epipleura slightly wider than outer glabrous one. Preepisternal plate narrow, 3× longer than wide, drop-like, without median longitudinal carina; median part slightly concave, bearing densely arranged small setiferous punctures, interstices without microsculpture; plate narrowly attached to metaventrite, posterior part slightly overlapping anterior margin of metaventrite (Fig. S2l). ***Metathorax*** (Fig. S2l). Metaventrite slightly longer than preepisternal elevation of mesothorax; its median portion not differing from lateral portion by slightly enlarged punctures. Punctation of median portion of metaventrite consisting of small dense setiferous punctures becoming slightly larger posteriad; interstices very narrow. Anterolateral ridge arcuately bending posteriad towards lateral margin of metaventrite, concave sublaterally. Median ridge of metaventrite reaching posterior margin of metaventrite. Lateral portion of femoral line present between anterolateral ridges and lateral sides of metaventrite, delimiting a triangular area. Anepisternum 8.0× longer than wide. ***Legs.*** Protibiae with shallow apical emargination on outer margin. ***Abdomen.*** Ventrites 1–3 with longitudinal ridges, ventrites 4–5 flat (Fig. S2m); posterior margin of ventrites 1–4 finely denticulate; abdominal ventrite 5 without emargination in both sexes. ***Male genitalia*** (Figs 4n– o). Aedeagus 0.71 mm long. Parameres 1.9× longer than phallobase, wide basally, continually narrowing apicad. Phallobase wide, as long as wide. Median lobe narrow basally, slightly constricted in apical 0.3, slightly widening and continually narrowing apicad. A pair of lateral subapical sclerites absent; apical sclerite narrow. Gonopore distinct, large. Median portion of sternite 9 shallowly angular.

Diagnosis. *Sacosternum laselva* sp. nov. is very similar to *S. lebbinorum* Fikáček & Short, 2010 in the form of the prosternum (with lateral extensions; Fig. S2k, arrow), the lateral part of metaventrite (with the triangular area; Fig. S2l, arrow), the form of mentum (widely pentagonal) and the form of the mesoventral plate (narrowly elongate; Fig. S2i, l) and the apex of the median lobe (without lateral sclerites; Fig. S2n, o). It also keys to *S. lebinnorum* in the identification key by Fikáček & Short (2010). It can be distinguished from *S. lebbinorum* by the form of the median lobe (bottle-shaped, i.e. wide basally and with apical narrow part in *S. laselva*, narrow from base to apex in *S. lebinnorum*), the presence of gonopore (indistinct in *S. lebbinorum*), and the apical sclerite of the median lobe very narrow at apex (widely triangular in *S. lebbinorum*). The new species most likely does not overlap with the range of *S. lebbinorum* which is restricted to northern South America.

Etymology. The species is named after the La Selva Biological Station where the type specimens were collected. Noun in apposition.

Biology. *Sacosternum laselva* was among the most common guests in *E. burchellii* refuse deposits (Fig. S2d) and was additionally collected in one *E. hamatum* deposit (Appendix S1). We also found it infrequently in *E. burchellii* emigrations (von Beeren, Blüthgen, et al., 2021). A single specimen was observed in a laboratory nest, where it had few, non-aggressive contacts to host army ants (von Beeren, Brückner, et al., 2021). It did not chemically resemble the nestmate recognition cues of the ants, and overall rather represented a non-specialized guest with a protective morphology (von Beeren, Brückner, et al., 2021).

Distribution. So far only known from the type locality, the La Selva Biological Station in Costa Rica.

PCR primer combinations used in this study.

Forward and reverse primers to amplify *COI* are given for each species. Bold letters highlight those primer combinations that amplified *COI* most reliably. Annealing temperatures varied between 45°C and 62°C. Primer sequences have been published previously (Folmer et al., 1994; Hajibabaei et al., 2005, 2006; Hebert et al., 2004; Hoenle et al., 2019; Meyer, 2003; von Beeren, Blüthgen, et al., 2021).

Species	Forward primer/reverse primer
***Achenomorphus* CVB04**	**LCO1490/HCO2198**
***Acrotrichis* CVB14**	**MLepF1/LepR1**
***Acrotrichis* CVB15**	**LCO1490/HCO2198**
***Acrotrichis* CVB16**	**LCO1490/HCO2198;** MLepF1/LepR1
***Acrotrichis* CVB17**	**MLepF1/LepR1**
***Acrotrichis* CVB18**	**LCO1490/HCO2198;** MLepF1/LepR1
***Acrotrichis* CVB19**	**LCO1490/HCO2198;** MLepF1/LepR1; dgLCO1490/CrematoR1
***Acrotrichis* CVB51**	**LCO1490/HCO2198**
**aff. *Pridonius* CVB09**	dgLCO1490/dgHCO2198; LCO1490/HCO2198; **MLepF1/LepR1**
**aff. *Pridonius* CVB70**	dgLCO1490/dgHCO2198; dgLCO1490/CrematoR1; LCO1490/HCO2198; **MLepF1/LepR1**
**aff. *Tetradonia* CVB50**	**LCO1490/HCO2198**
***Aleochara* CVB30**	**LCO1490/HCO2198**
**Aleocharinae CVB31**	**LCO1490/dgHCO2198**
**Aleocharinae CVB32**	**LCO1490/HCO2198;** MLepF1/LepR1
**Aleocharinae CVB33**	**LCO1490/dgHCO2198; MLepF1/LepR1**
**Aleocharinae CVB35**	**LCO1490/HCO2198**; LCO1490/dgHCO2198
**Aleocharinae CVB36**	**LCO1490/HCO2198**; LCO1490/dgHCO2198; MLepF1/LepR1; dgLCO1490/CrematoR1
**Aleocharinae CVB37**	**LCO1490/HCO2198**
**Aleocharinae CVB43**	**LCO1490/HCO2198;** LCO1490/dgHCO2198; dgLCO1490/CrematoR1
**Aleocharinae CVB44**	LCO1490/HCO2198; **MLepF1/LepR1**
**Aleocharinae CVB46**	**LCO1490/dgHCO2198; MLepF1/LepR1**
**Aleocharinae CVB47**	**LCO1490/HCO2198;** LCO1490/dgHCO2198
**Aleocharinae CVB48**	**LCO1490/HCO2198; MLepF1/LepR1**
**Aleocharinae CVB49**	**LCO1490/HCO2198**
**Aleocharinae CVB54**	**LCO1490/HCO2198**
**Aleocharinae CVB55**	**LCO1490/HCO2198; MLepF1/LepR1**
***Apalonia* CVB34**	**LCO1490/HCO2198;** LCO1490/dgHCO2198
**Cantharidae CVB62**	**LCO1490/HCO2198**
Cephaloplectus mus	**dgLCO1490/dgHCO2198**
Cercyon pohli	**LCO1490/HCO2198**
Cheilister* cf. *lucidulus	**dgLCO1490/CrematoR1**
***Clientister* CVB95**	**LCO_Ecc2/ HCO2198**

Clinidium erwini	**LCO1490/HCO2198**
**Coleoptera CVB24**	**LCO1490/dgHCO2198**
**Coleoptera CVB25**	**LCO1490/HCO2198**
Coproporus **CVB11**	**LCO1490/HCO2198; LCO1490/dgHCO2198**; dgLCO1490/CrematoR1; MLepF1/LepR1
Coproporus **CVB12**	**LCO1490/HCO2198;** MLepF1/LepR1
Coproporus **CVB71**	**LCO1490/HCO2198;** LCO1490/dgHCO2198
Coproporus **CVB81**	**LCO1490/HCO2198**
Daptesister pilosus	**LCO1490/HCO2198**
**Discheramocephalini CVB13**	**LCO1490/HCO2198**
**Discheramocephalini CVB20**	**LCO1490/HCO2198**
Dissochaetus confusus	**LCO1490/HCO2198**
Dissochaetus hetschkoi	**LCO1490/HCO2198;** LCO1490/dgHCO2198
Ecitodonia **CVB65**	**LCO1490/HCO2198;** MLepF1/LepR1; LCO1490/dgHCO2198; MLepF1/LepR1
Ecitomorpha **cf.** ***breviceps***	**LCO1490/HCO2198**
Ecitomorpha **cf.** ***nevermanni***	**LCO1490/HCO2198**
Ecitophya simulans	**LCO1490/HCO2198**
Ecitopora **CVB63**	**LCO1490/HCO2198;** LCO1490/dgHCO2198; dgLCO1490/HCO2198; MLepF1/LepR1
Euclasea **CVB66**	**MLepF1/LepR1**
Euxenister caroli	**LCO1490/HCO2198**
**False-Lomechusini CVB67**	**LCO1490/HCO2198**
**False-Lomechusini CVB68**	**LCO1490/HCO2198**
Geotrochopsis pubescens	**MLepF1/LepR1**
Limulodes **CVB64**	**LCO1490/dgHCO2198**
Lithocharis **cf.** ***discoidalis***	**LCO1490/HCO2198;** LCO1490/dgHCO2198; LepF1/LepR1; MLepF1/LepR1; dgLCO1490/CrematoR1
Lithocharis **CVB05**	**LCO1490/HCO2198;** MLepF1/LepR1
Lithocharis **CVB07**	**LCO1490/HCO2198**
Lithocharis **CVB08**	**LCO1490/HCO2198**
**Medonina CVB52**	**LCO1490/HCO2198**
**Medonina CVB53**	**LCO1490/HCO2198**
**Meronera CVB42**	**LCO1490/HCO2198;** LCO1490/dgHCO2198; MLepF1/LepR1
Myrmedonota **CVB38**	**LCO1490/HCO2198;** MLepF1/LepR1
Myrmedonota **CVB39**	**LCO1490/HCO2198; MLepF1/LepR1**
Myrmedonota **CVB41**	**LCO1490/HCO2198;** LCO1490/dgHCO2198; dgLCO1490/dgHCO2198; dgLCO1490/CrematoR1; MLepF1/LepR1
Myrmedonota **CVB45**	**LCO1490/HCO2198;** MLepF1/LepR1
Myrmedonota **CVB69**	**dgLCO1490/CrematoR1**
Neocolonides howdeni	**LCO_Ecc_Nym1/HCO2198**
Onthophagus limonensis	**LCO1490/HCO2198**
Phelister **CVB85**	**LCO_Ecc2/HCO2198; MLepF1/LepR1**

***Plagiogramma* CVB61**	**LCO_Ecc2/HCO_Ecc1**
Plagiogramma schmidti	**Amplification failed**
Psalidister furcatus	**LCO_Ecc2/HCO2198; LCO_Ecc2/HCO_Ecc1**
**Ptilodactylidae CVB22**	**MLepF1/LepR1**
Sacosternum laselva	**LCO1490/HCO2198; MLepF1/LepR1**
**Scraptiidae CVB29**	**LCO1490/HCO2198**
**Staphylinidae CVB23**	**LCO1490/HCO2198**
Symphilister* cf. *hamati	**LCO1490/HCO2198;** MLepF1/LepR1
Synoditulus debilis	**LCO_Ecc2/HCO2198;** LCO_Ecc2/HCO_Ecc1; MLepF1/LepR1
**Tenebrionidae CVB21**	**MLepF1/LepR1**
***Tetradonella* CVB40**	**LCO1490/HCO2198;** LepF1/LepR1
Tetradonia* cf. *marginalis	**LCO1490/HCO2198**
Tetradonia laticeps	**LCO1490/HCO2198**
**Trechinae CVB28**	**LCO1490/HCO2198;** MLepF1/LepR1
Trichoreninus geminus	**LCO_Ecc2/HCO2198**
***Vatesus* cf. *clypeatus* CVB72**	**LCO1490/HCO2198**
**Xantholinini CVB10**	**LCO1490/HCO2198**

Supplemental reference list

Akre, R. D., & Rettenmeyer, C. W. (1966). Behavior of Staphylinidae associated with army ants (Formicidae: Ecitonini). *Journal of the Kansas Entomological Society*, *39*(4), 745–782.

Arriaga-Varela, E., Seidel, M., Deler-Hernández, A., Senderov, V., & Fikáček, M. (2017). A review of the *Cercyon* Leach (Coleoptera, Hydrophilidae, Sphaeridiinae) of the Greater Antilles. *ZooKeys*, *681*, 39–93. https://doi.org/10.3897/zookeys.681.12522

Arriaga-Varela, E., Sỳkora, V., & Fikáček, M. (2021). Molecular phylogeny of Megasternini terrestrial water scavenger beetles (Hydrophilidae) reveals repeated continental interchange during Paleocene-Eocene thermal maximum. *Systematic Entomology*, *46*(3), 570–591. https://doi.org/10.1111/syen.12476

Bell, R. T., & Bell, J. R. (2009). Rhysodine beetles (Insecta: Coleoptera: Carabidae): new species, new data III. *Annals of Carnegie Museum*, *78*(1), 45–77. https://doi.org/10.2992/007.078.0104

Caterino, M. S., & Tishechkin, A. K. (2020). Recognition and revision of the *Phelister blairi* group (Histeridae, Histerinae, Exosternini). *ZooKeys*, *1001*, 1–154. https://doi.org/10.3897/zookeys.1001.58447

Chani-Posse, M. R., Brunke, A. J., Chatzimanolis, S., Schillhammer, H., & Solodovnikov, A. (2018). Phylogeny of the hyper-diverse rove beetle subtribe Philonthina with implications for classification of the tribe Staphylinini (Coleoptera: Staphylinidae). *Cladistics*, *34*(1), 1–40. https://doi.org/10.1111/cla.12188

Dégallier, N. (1998). Coleoptera Histeridae Hetaeriinae: Description de nouveaux taxons, désignation de lectotypes et notes taxonomiques. *Bonner Zoologische Beitrage*, *47*, 345–379.

Fikáček, M., & Short, A. E. (2010). A revision of the Neotropical genus *Sacosternum* Hansen (Hydrophilidae: Sphaeridiinae: Megasternini). *Zootaxa*, *2538*(1), 1–37. https://doi.org/10.11646/zootaxa.2538.1.1

Folmer, O., Black, M., Hoeh, W., Lutz, R., & Vrijenhoek, R. (1994). DNA primers for amplification of mitochondrial *cytochrome c oxidase subunit I* from diverse metazoan invertebrates. *Molecular Marine Biology and Biotechnology*, *3*(5), 294–299.

Gnaspini, P. (1993). Brazilian Cholevidae (Coleoptera), with emphasis on cavernicolous species. III. *Dissochaetus* larvae, with description of a new feature. *Revista Brasileira de Entomologia*, 37(3), 545-553.

Hajibabaei, M., dewaard, J. R., Ivanova, N. V., Ratnasingham, S., Dooh, R. T., Kirk, S. L., Mackie, P. M., & Hebert, P. D. (2005). Critical factors for assembling a high volume of DNA barcodes. *Philosophical Transactions of the Royal Society B: Biological Sciences*, *360*(1462), 1959–1967. https://doi.org/10.1098/rstb.2005.1727

Hajibabaei, M., Janzen, D. H., Burns, J. M., Hallwachs, W., & Hebert, P. D. N. (2006). DNA barcodes distinguish species of tropical Lepidoptera. *Proceedings of the National Academy of Sciences of the United States of America*, *103*(4), 968–971. https://doi.org/10.1073/pnas.0510466103

Hebert, P. D. N., Penton, E. H., Burns, J. M., Janzen, D. H., & Hallwachs, W. (2004). Ten species in one: DNA barcoding reveals cryptic species in the neotropical skipper butterfly *Astraptes fulgerator*. *Proceedings of the National Academy of Sciences of the United States of America*, *101*(41), 14812–14817. https://doi.org/10.1073/pnas.0406166101

Helava, J. V. T., Howden, H. F., & Ritchie, A. J. (1985). A review of the new world genera of the myrmecophilous and termitophilous subfamily Hetaeriinae (Coleoptera: Histeridae). *Sociobiology*, *10*(2), 127–386.

Hoenle, P. O., Blüthgen, N., Brückner, A., Kronauer, D. J., Fiala, B., Donoso, D. A., Smith, M. A., Ospina Jara, B., & von Beeren, C. (2019). Species-level predation network uncovers high prey specificity in a Neotropical army ant community. *Molecular Ecology*, *28*(9), 2423–2440. https://doi.org/10.1111/mec.15078

Irmler, U. (1981). Neue Arten der Gattung *Mimogonia* Coiffait (1978) aus der Neotropis (Coleoptera, Staphylinidae). *Entomologische Blätter*, *77*(3), 143–152.

Irmler, U. (2016). A new genus and new species of Neotropical Thoracophorini (Coleoptera: Staphylinidae: Osoriinae). *Bonn Zoological Bulletin*, *64*(2), 109–116.

Jacobson, H. R., & Kistner, D. H. (1998). A redescription of the myrmecophilous genus *Tetradonia* and a description of a new, closely related, free living genus *Tetradonella* (Coleoptera: Staphylinidae). *Sociobiology*, *31*, 151–279.

Kistner, D. H., & Jacobson, H. R. (1990). Cladistic analysis and taxonomic revision of the ecitophilous tribe Ecitocharini with studies of their behavior and evolution (Coleoptera, Staphylinidae, Aleocharinae). *Sociobiology*, *17*, 333–480.

Kistner, D. H., & Mooney, R. R. (2011). Revision of the genus *Ecitopora* with descriptions of new species (Coleoptera: Staphylinidae). *Sociobiology.*, *58*(2), 269–307.

Kohlmann, B., & Solís, A. (2001). El género *Onthophagus* (Coleoptera: Scarabaeidae). *Giornale Italiano Di Entomologia*, *9*, 159–261.

Meyer, C. P. (2003). Molecular systematics of cowries (Gastropoda: Cypraeidae) and diversification patterns in the tropics. *Biological Journal of the Linnean Society*, *79*(3), 401–459. https://doi.org/10.1046/j.1095-8312.2003.00197.x

Navarrete-Heredia, J., Newton, A. F., Thayer, M. K., Ashe, J. S., & Chandler, D. (2002). *Guía ilustrada para los géneros de Staphylinidae (Coleoptera) de México. Illustrated guide to the genera of Staphylinidae (Coleoptera) of México.* Universidad de Guadalajara, Guadalajara, Mexico.

Peck, S. B. & Cook, J. (2017). A review of the small carrion beetle genus *Dissochaetus* Reitter (Coleoptera: Leiodidae: Cholevinae) of Central America. *Dugesiana*, 24(2), 91-119.

Peck, S. B., Gnaspini, P. & Newton A. F. (2020). Updated catalog and generic keys of the Leiodidae (Insecta: Coleoptera) of the Neotropical region ("Latin America": Mexico, the West Indies, and Central and South America). *Zootaxa*, 4741(1), 001-114.

Seevers, C. H. (1965). The systematics, evolution and zoogeography of staphylinid beetles, associated with army ants (Coleoptera, Staphylinidae). *Fieldiana Zoology*, *47*, 137–351.

Sharp, D. (1882). Insecta, Coleoptera.(Haliplidae, Dytiscidae, Gyrinidae, Hydrophilidae, Heteroceridae, Parnidae, Georissidae, Cyathoceridae). *Biologia Centrali-Americana*, *1*(2), 1–144.

Smetana, A. (1982). Revision of the subfamily Xantholininae of America north of Mexico (Coleoptera: Staphylinidae). *The Memoirs of the Entomological Society of Canada*, *114*(S120), 1–389.

Tishechkin, A. K. 2007. Phylogenetic revision of the genus *Mesynodites* (Coleoptera: Histeridae: Hetaeriinae) with description of new tribes, genera and species. *Sociobiology 49: 1–167.*

Vasilikopoulos, A., Balke, M., Kukowka, S., Pflug, J. M., Martin, S., Meusemann, K., Hendrich, L., Mayer, C., Maddison, D. R., & Niehuis, O. (2021). Phylogenomic analyses clarify the pattern of evolution of Adephaga (Coleoptera) and highlight phylogenetic artefacts due to model misspecification and excessive data trimming. *Systematic Entomology*, *46*(4), 991–1018. https://doi.org/10.1111/syen.12508

von Beeren, C., Blüthgen, N., Hoenle, P. O., Pohl, S., Brückner, A., Tishechkin, A. K., Maruyama, M., Brown, B. V., Hash, J. M., Hall, W. E., & Kronauer, D. J. C. (2021). A remarkable legion of guests: Diversity and host specificity of army ant symbionts. *Molecular Ecology*, *30*(20), 5229–5246. https://doi.org/10.1111/mec.16101

von Beeren, C., Brückner, A., Hoenle, P. O., Ospina-Jara, B., Kronauer, D. J., & Blüthgen, N. (2021). Multiple phenotypic traits as triggers of host attacks towards ant symbionts: Body size, morphological gestalt, and chemical mimicry accuracy. *Frontiers in Zoology*, *18*(1), 1–18. https://doi.org/10.1186/s12983-021-00427-8

von Beeren, C., Brückner, A., Maruyama, M., Burke, G., Wieschollek, J., & Kronauer, D. J. C. (2018). Chemical and behavioral integration of army ant-associated rove beetles–a comparison between specialists and generalists. *Frontiers in Zoology*, *15*(1), 8. https://doi.org/10.1186/s12983-018-0249-x

von Beeren, C., Maruyama, M., & Kronauer, D. J. C. (2016a). Community sampling and integrative taxonomy reveal new species and host specificity in the army ant-associated beetle genus *Tetradonia* (Coleoptera, Staphylinidae, Aleocharinae). *PLoS ONE*, *11*, e0165056. https://doi.org/10.1371/journal.pone.0165056

von Beeren, C., Maruyama, M., & Kronauer, D. J. C. (2016b). Cryptic diversity, high host specificity and reproductive synchronization in army ant-associated *Vatesus* beetles. *Molecular Ecology*, *25*, 990–1005. https://doi.org/10.1111/mec.13500

Yamamoto, S. (2021). Tachyporinae revisited: phylogeny, evolution, and higher classification based on morphology, with recognition of a new rove beetle subfamily (Coleoptera: Staphylinidae). *Biology, 10(4), 323.* https://doi.org/10.3390/biology10040323

Żyla, D., Bogri, A., Heath, T. A., & Solodovnikov, A. (2021). Total-evidence analysis resolves the phylogenetic position of an enigmatic group of Paederinae rove beetles (Coleoptera: Staphylinidae). *Molecular Phylogenetics and Evolution*, *157*, 107059. https://doi.org/10.1016/j.ympev.2020.107059

Żyla, D., Yamamoto, S., & Jenkins Shaw, J. (2019). Total-evidence approach reveals an extinct lineage of Paederinae rove beetles from Cretaceous Burmese amber. *Palaeontology*, *62*(6), 935–949. https://doi.org/10.5061/dryad.37jj6t9
