# Supplementary material for: Army ant middens – Home and nursery of a diverse beetle fauna
Source: Ecol Evol. 2023 Sep 19;13(9):e10451. doi: 10.1002/ece3.10451 (PMC10509147; doi:10.1002/ece3.10451)

Appendix 3. RAxML tree based on GTR GAMMA as substitution model of 414 *COI* sequences of refuse-visiting beetles. We used the RAxML plugin for GENEIOUS PRIME (version 2020.2.1) to generate the tree. Terminal labels provide the following information: sample ID, BOLD Process ID, GenBank accession number, species name. The RAxML tree was rooted using the following outgroup: *Hydroscapha takahashii* (GenBank accession number: MT132896), a beetle of the suborder Myxophaga. The scale bar indicates expected nucleotide substitutions per site as inferred by the RAxML algorithm. Bootstrap support values are shown at nodes (1,000 repetitions). Colors highlight the different species.

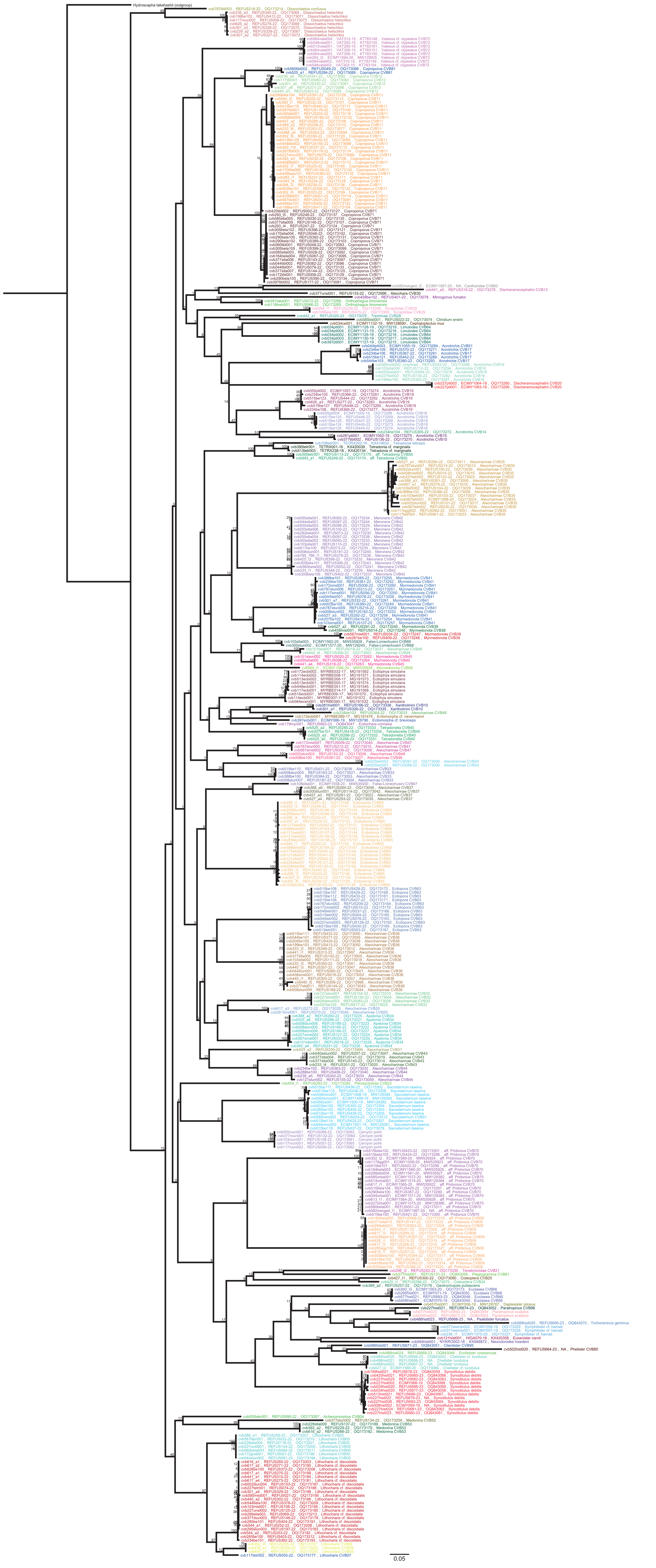

Supplement: Supplementary file 3 — Appendix S3. [file ECE3-13-e10451-s002.pdf]
